# Small-Molecule Immunosuppressive Drugs and Therapeutic Immunoglobulins Differentially Inhibit NK Cell Effector Functions *in vitro*

**DOI:** 10.3389/fimmu.2019.00556

**Published:** 2019-03-27

**Authors:** Amandine Pradier, Maria Papaserafeim, Ning Li, Anke Rietveld, Charlotte Kaestel, Lyssia Gruaz, Cédric Vonarburg, Rolf Spirig, Gisella L. Puga Yung, Jörg D. Seebach

**Affiliations:** ^1^Division of Immunology and Allergy, University Hospitals and Medical Faculty, Geneva, Switzerland; ^2^CSL Behring AG, Bern, Switzerland

**Keywords:** human NK cells, immunosuppression, cytotoxicity and ADCC, IVIg, methylprednisolone, mycophenolic acid, cyclosporine A and tacrolimus, everolimus

## Abstract

Small-molecule immunosuppressive drugs (ISD) prevent graft rejection mainly by inhibiting T lymphocytes. Therapeutic immunoglobulins (IVIg) are used for substitution, antibody-mediated rejection (AbMR) and HLA-sensitized recipients by targeting distinct cell types. Since the effect of ISD and IVIg on natural killer (NK) cells remains somewhat controversial in the current literature, the aim of this comparative study was to investigate healthy donor's human NK cell functions after exposure to ISD and IVIg, and to comprehensively review the current literature. NK cells were incubated overnight with IL2/IL12 and different doses and combinations of ISD and IVIg. Proliferation was evaluated by ^3^[H]-thymidine incorporation; phenotype, degranulation and interferon gamma (IFNγ) production by flow cytometry and ELISA; direct NK cytotoxicity by standard ^51^[Cr]-release and non-radioactive DELFIA assays using K562 as stimulator and target cells; porcine endothelial cells coated with human anti-pig antibodies were used as targets in antibody-dependent cellular cytotoxicity (ADCC) assays. We found that CD69, CD25, CD54, and NKG2D were downregulated by ISD. Proliferation was inhibited by methylprednisolone (MePRD), mycophenolic acid (MPA), and everolimus (EVE). MePRD and MPA reduced degranulation, MPA only of CD56^bright^ NK cells. MePRD and IVIg inhibited direct cytotoxicity and ADCC. Combinations of ISD demonstrated cumulative inhibitory effects. IFNγ production was inhibited by MePRD and ISD combinations, but not by IVIg. In conclusion, IVIg, ISD and combinations thereof differentially inhibit NK cell functions. The most potent drug with an effect on all NK functions was MePRD. The fact that MePRD and IVIg significantly block NK cytotoxicity, especially ADCC, has major implications for AbMR as well as therapeutic strategies targeting cancer and immune cells with monoclonal antibodies.

## Introduction

NK cells exert both effector and regulatory functions mediated by direct cytotoxicity, death receptor/ligand-mediated cytotoxicity, CD16 (FcγRIIIA)-mediated antibody-dependent cellular cytotoxicity (ADCC), and cytokine secretion, in particular interferon gamma (IFNγ) and tumor necrosis factor (TNF). In addition to their important role in antitumor and antiviral immune responses (1, 2), for example controlling post-transplant viral infections and lymphoproliferative disease, NK cells are able to shape immune responses following transplantation. In HLA-mismatched hematopoietic stem cell transplantation, alloreactive NK cells were shown to have beneficial anti-leukemic effects, and may prevent graft- vs.-host disease by killing host dendritic cells (3, 4). NK cells are also promising candidates for cell-based leukemia and cancer immunotherapies (5). Whereas, NK cells are clearly important in xenograft rejection (6), less is known about NK alloreactivity in solid organ transplantation (SOT) (7–11). A role of NK cell in antibody-mediated rejection (AbMR) was first demonstrated by the presence of NK cell transcripts and infiltration in biopsies of kidney transplant recipients positive for donor-specific antibodies (12). Thus, NK cells enhance alloresponses and contribute to antibody-mediated heart and kidney allograft vasculopathy most likely by inducing endothelial cell damage via ADCC (13–17). In contrast, NK cells also seem to be able to induce long-term allograft tolerance (11).

As long as induction of tolerance cannot be routinely achieved, pharmacological immunosuppression is still required to prevent rejection in transplantation medicine. However, small-molecule immunosuppressive drugs (ISD) currently used in the clinic are associated with serious side effects, including drug toxicity, infections and tumorigenesis. This is the reason why immunosuppressive regimens are regularly refined according to the clinical needs and the growing basic knowledge of the molecular and cellular effects of ISD on the immune system. ISD target different intracellular signaling pathways downstream of the immune synapse (18–20): cyclosporine A (CsA) and tacrolimus (TAC) are calcineurin-inhibitors; mycophenolic acid (MPA), the active metabolite of mycophenolate mofetil (MMF), inhibits cell proliferation; everolimus (EVE) or rapamycin (RAPA) are mTOR-inhibitors; and corticosteroids (methylprednisolone, MePRD) block the transcription of a broad range of proinflammatory genes at different levels. ISD were developed to inhibit T cell-mediated graft rejection and are often used in combination; however, their effect on NK cells is less clear because of partially conflicting evidence stemming from different experimental approaches.

Polyspecific intravenous immunoglobulin preparations (IVIg) are used both, in SOT and hematopoietic stem cell transplantation, as a treatment for post-transplant infections; as a prevention for graft-vs.-host disease; and as substitution for secondary post-transplant hypogammaglobulinemia. Furthermore, IVIg play a role in protocols for highly HLA-sensitized SOT recipients, and in the management of antibody-mediated rejection (21). A considerable number of studies have addressed the mechanisms of action of IVIg on different immune cells (22, 23); however, the effect of IVIg on NK cells remains to some degree controversial.

The aim of this comprehensive and comparative study was to determine the impact of ISD and IVIg, at doses used in daily clinical practice, on the proliferation, phenotype, direct cytotoxicity, ADCC and cytokine production of healthy donor's peripheral blood NK cells. Our results elucidate some of the controversial data, and provide new evidence, in particular on ADCC, ISD combinations, and directly comparing ISD with IVIg, that allow in conjunction with a thorough review of the literature to draw firmer conclusions on the effect of ISD and IVIg on NK cell functions.

## Materials and Methods

### ISD and IVIg

ISD and IVIg concentrations were chosen in order to represent therapeutic serum levels, i.e., peak and trough levels, and a 1:10 dilution of the trough levels: CsA (0.01-1 μg/ml), TAC (0.001–0.1 μg/ml), MPA (0.05–5 μg/ml), EVE (0.001–0.1 μg/ml), MePRD (0.005-5 μg/ml) were dissolved in ethanol (EtOH); whereas IVIg (0.25–25 mg/ml) were used in a carrier solution (vehicle) containing stabilizer (250 mM L-proline and 20 μg/ml polysorbate 80, CSL Behring). When used combined, the following concentrations were used: CsA (0.1 μg/ml); TAC (0.01 μg/ml); MPA (5 μg/ml); EVE (0.01 μg/ml), and MePRD (0.5 μg/ml).

### NK Cell Isolation and Culture

Blood samples were obtained from healthy donors at the Geneva blood bank with approval of the local ethical committee (N° 08–133 and 13–149). NK cells were isolated using magnetic negative selection (Miltenyi) with a purity of routinely >95% as described previously (24). Purified NK cells were cultured in AIMV medium containing 2% HEPES buffer, and incubated in the presence or not of the active forms of CsA, TAC, MPA, EVE, and MePRD alone or in combinations; IVIg was tested in separate experiments.

### NK Cell Survival

Freshly purified human NK cells were incubated overnight in the presence of absence of different doses of ISD or IVIg. After 18 h of incubation, the cells were labeled with 7-amino-actinomycin D (7AAD) (Sigma) at a final concentration of 1 μg/ml and FACS analysis was performed.

### Proliferation Assay

Freshly purified human NK cells (0.05 × 10^6^/200 μl) or the corresponding non-NK cell fraction stemming from the isolation procedure were incubated for 5d with human recombinant IL2 (Novartis, 50 U/ml), in the absence of IL12, a protocol commonly used for NK cell expansion *in vitro* (25, 26) plus ISD or IVIg in 96-well-plates in triplicates. At the end of the experiment the cells were pulsed with 0.5 μCi/well during 19 h and ^3^[H]-thymidine (PerkinElmer) incorporation was measured using a scintillation beta-counter (Perkin Elmer 2450 Microplate counter, MicroBeta 2 TM).

### NK Cell Characterization

Peripheral blood mononuclear cells (PBMC) were incubated with IL2 alone (50 U/ml, control) or with individual combinations of CsA (0.1 μg/ml), TAC (0.01 μg/ml), MPA (5 μg/ml), EVE (0.01 μg/ml), and MePRD (0.5 μg/ml) for 24 h, when possible using the same donor in each experiment. NK cell phenotype was determined by direct staining with antibodies for CD3 (clone UCHT1), CD56 (clone AF12-7H3) and CD16 (clone 3G8); for NK activation markers (CD25, clone 2A3 and CD69, clone FN50); adhesion molecules (anti-CD54 clone HA58); NK receptors including C-type lectins (NKG2A, NKG2C, and NKG2D, clones 131411, 1345591, and BAT221, respectively); and natural cytotoxicity receptors (NCRs: NKp30, NKp44, and NKp46, clones AF29-4D12, 2.29, and 9E2, respectively), by FACS Calibur (BD Biosciences) or Attune cytometer (Life Technologies) using isotype control antibodies. Propidium iodide (PI) (Sigma) or 7AAD staining was used to exclude dead cells. Levels of surface expression are shown as the geometric mean fluorescence intensity ratios (MFIR) (27).

### Analysis of Degranulation by CD107a Expression and Intracellular IFNγ Staining

CD107a surface expression as a marker for degranulation and intracellular IFNγ positive cells were detected according to Alter et al with minor modifications (28). Isolated NK cells were incubated overnight with a combination of IL2 and IL12 (R&D Systems) (50 U/ml and 0.5 ng/ml, respectively) to obtain measurable amounts of intracellular IFNγ production in the presence of absence of different doses of ISD or IVIg. After 18 h of incubation, the cells were labeled with anti-CD107a (eBioscience); and further stimulated by the addition of the K562 cells in a ratio of 1:1 for 1 h at 37°C after which Golgistop™ (BD Biosciences) was added for 2 additional hours at 37°C. ISD were present throughout the entire assay. Intracellular staining with anti-IFNγ antibody (Biolegend) was carried out following the manufacturer's instructions.

### Cytotoxicity Assays

Purified human NK cells were used as effector cells in the presence of ISD in standard ^51^[Cr]-release cytotoxicity assays against the NK target cell line K562 as described previously (24), with minor modifications. NK cells were incubated overnight with IL2 and IL12 (50 U/ml and 0.5 ng/ml, respectively) in addition to ISD or IVIg without subsequent washing. K562 cells were labeled with ^51^[Cr] (Hartmann Analytica) and used at E:T ratios starting at 10:1. For ADCC, porcine endothelial cells of D haplotype (PED) (29) were used as targets at E:T ratios starting at 25:1, following pre-incubation with heat-inactivated human serum (10%) containing human-anti-pig natural antibodies (30). Incubation of NK cells with medium alone without serum was used as direct cytotoxicity control. For IVIg experiments, a non-radioactive DELFIA cytotoxicity assay was used (31). Target cells were labeled with a fluorescence enhancing ligand (BADTA) and co-cultured with NK cells for 2 h. The supernatants were then measured by time-resolved fluorometry (EnVision 2014 Multilabel reader, PerkinElmer). The percentage of specific lysis was calculated as described before (30).

### Determination of Secreted IFNγ by ELISA

Purified NK cells were incubated overnight with IL2 (50 U/ml) and IL12 (0.5 ng/ml) in the presence or absence of different doses of ISD or IVIg. The cells were incubated for additional 3 h with K562 cells at an E:T ratio 1:1. Supernatants were collected and IFNγ secretion was quantified using the Human IFNγ DuoSet kit (R&D Systems) according to the manufacturer's instructions. Plates were analyzed using a VersaMax microplate reader (Molecular Devices). All experiments were performed in triplicates.

### Statistical Analysis

GraphPad Prism software (version 7.02) was used for all statistics. Significance between groups was tested by one-way ANOVA for matched data followed by Dunnett's multiple comparisons testing using either IL2; IL2/IL12; or sera conditions as reference for proliferation; direct cytotoxicity, degranulation and IFNγ; or ADCC, respectively. The number of experiments is indicated in the Figure and Table legends, three experiments were only considered sufficient when the results were unequivocal. Additionally, paired *t*-tests were used. *p* < 0.05 were considered significant.

## Results

### Immunosuppressive Drugs Inhibit NK Cell and T Cell Proliferation to a Similar Extent

**Figure 1:**
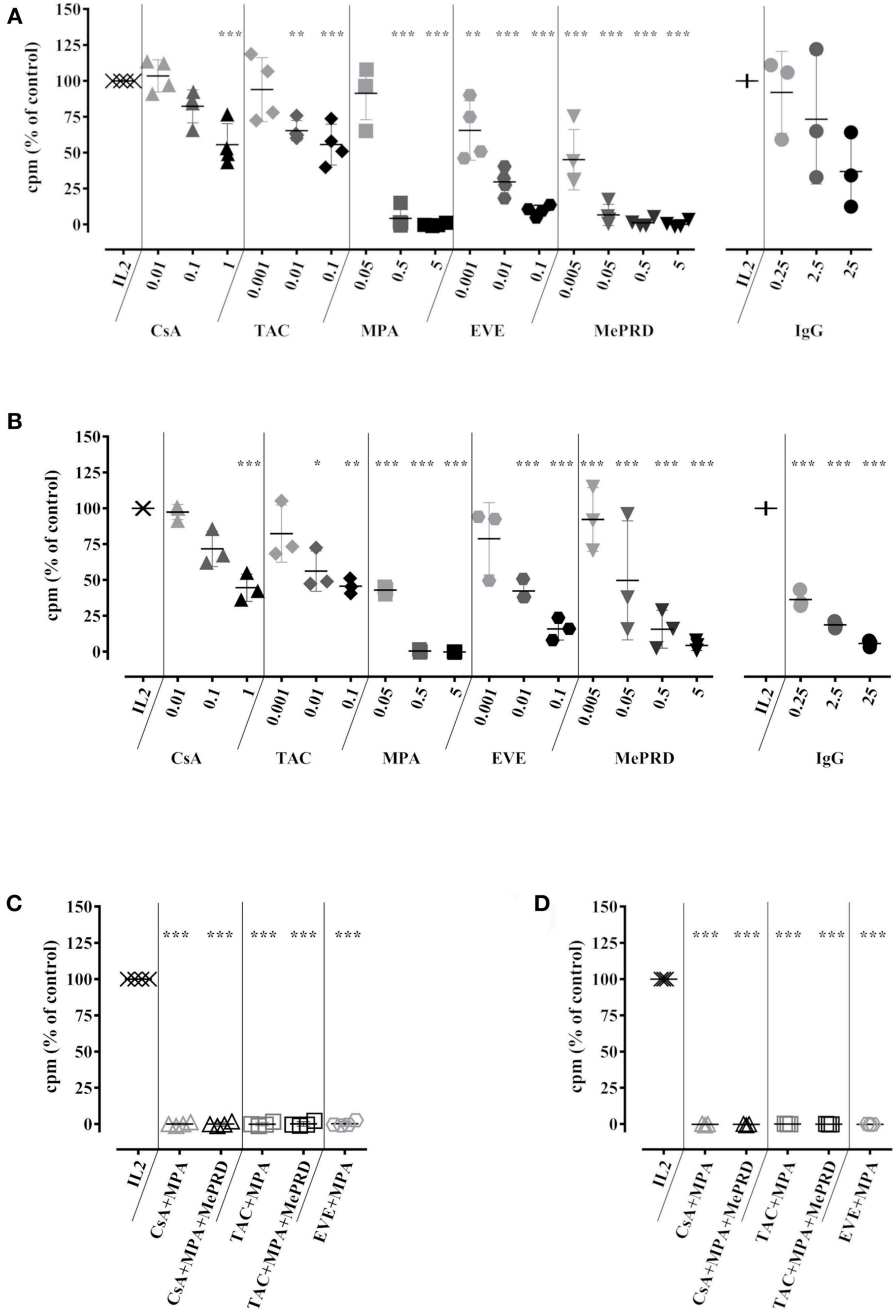
Immunosuppressive drugs inhibit NK cell and T cell proliferation to a similar extent. Human NK cells isolated from PBMC **(A)**, or the non-NK cell fraction containing T cells **(B)** were incubated with IL2 (50 U/ml) in the presence or not of CsA (0.01–1 μg/ml), TAC (0.001–0.1 μg/ml), MPA (0.05–5 μg/ml), EVE (0.001–0.1 μg/ml) MePRD (0.005–5 μg/ml) and IVIg (0.25–25 mg/ml). Data for ISD combinations on isolated NK cells, or the non-NK cell fraction are shown in **(C,D)**, respectively. When used in combination, the following concentrations of ISD were used: CsA (0.1 μg/ml); TAC (0.01 μg/ml); MPA (5 μg/ml); EVE (0.01 μg/ml), and MePRD (0.5 μg/ml). After 5d, ^3^[H]-thymidine was added and cells were incubated 19 h before measuring cellular proliferation by ^3^[H]-incorporation. Controls included NK cells stimulated with IL2 and carrier/vehicle solutions containing ethanol (EtOH). Data are presented as mean % of IL2 controls ± SD. Symbols represent individual experiments (*n* = 3 or 4). Statistical analysis was performed using one-way ANOVA with Dunnett's Multiple Comparison Test as post-test vs. IL2 control. *p < 0.05, **p < 0.01, ***p < 0.005.

NK cell proliferation was assessed in 5d IL2 stimulation assays and compared with T cell proliferation using NK-depleted PBMC. Different concentrations of ISD were used in order to simulate trough and peak drug levels present in the serum of patients under different ISD regimens. These concentrations were not toxic for NK cells (Figure S1). A dose-dependent inhibition of NK cell proliferation was observed for all ISD and clinically relevant concentrations (Figure 1A). MPA and MePRD were the most potent inhibitors. Clinically used combinations of ISD had a cumulative effect and completely abrogated NK cell proliferation (Figure 1C). The inhibitory effect of ISD on NK cell proliferation was similar to that on T cell proliferation for a single drug and combinations (Figures 1B,D respectively).

Treatment with increasing doses of IVIg tended to decrease NK cell proliferation proportionally, but without statistical significance (*p* = 0.0745) (Figures 1A,B). In contrast, IVIg induced a dose-dependent reduction of T cell proliferation, as previously reported (32, 33).

Thus, ISD inhibit NK cell proliferation without inducing drug-related toxicity, combinations of ISD exhibited a cumulative effect, resulting in complete inhibition, while IVIg had no significant effect.

### Effect of Immunosuppressive Drugs on the Expression of NK Cell Markers and Receptors

**Figure 2:**
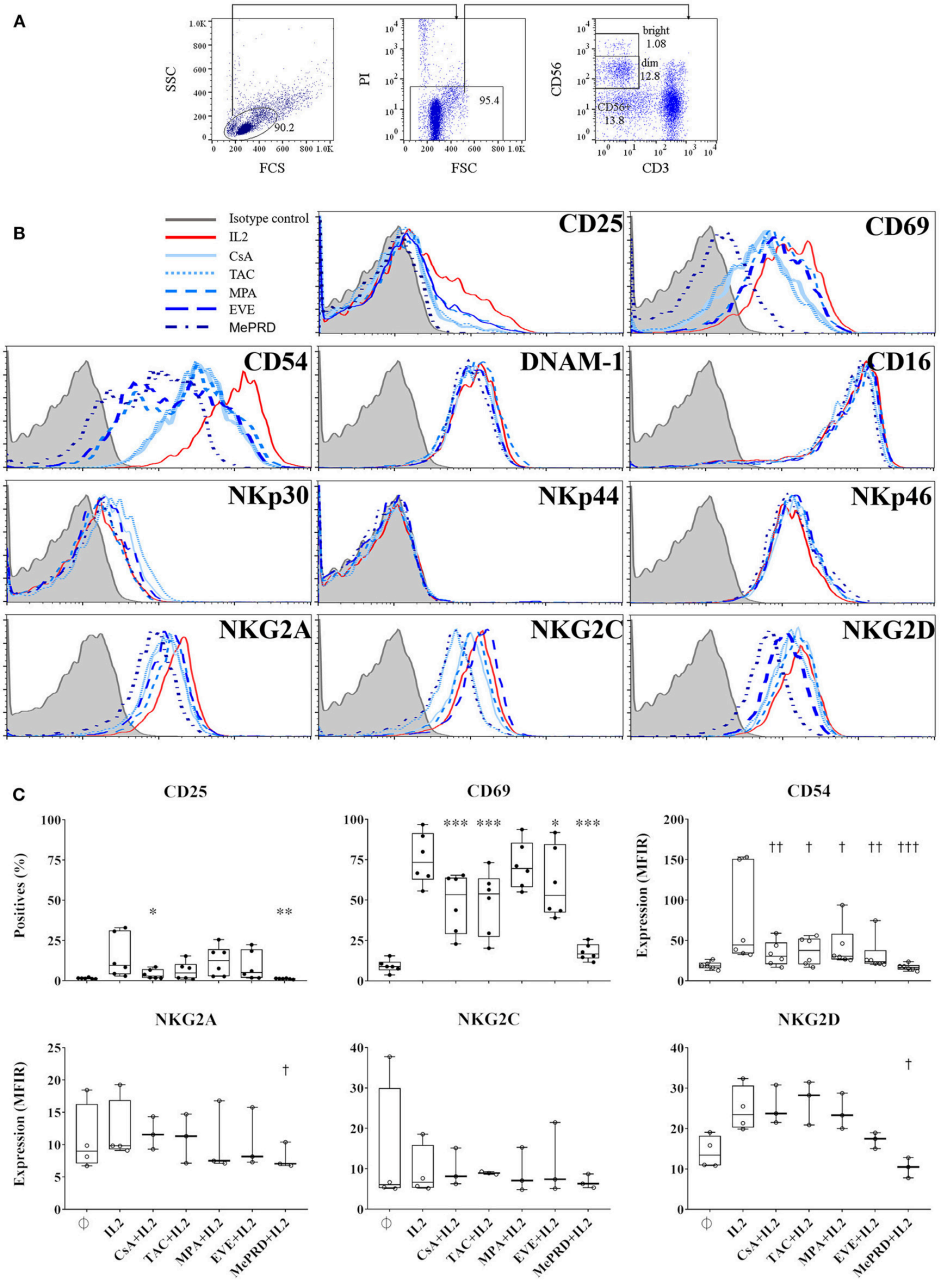
Inhibition of NK cell marker and receptor expression by MePRD and differential effect of calcineurin inhibitors and EVE. PBMC were incubated with or without IL2 (50 U/ml) (control); with IL2 (50 U/ml) plus CsA (0.1 μg/ml), TAC (0.01 μg/ml), MPA (5 μg/ml), EVE (0.01 μg/ml), or MePRD (0.5 μg/ml) for 24 h. NK cell marker and receptor expression was analyzed by FACS. The gating strategy is shown **(A)**, and representative histogram plots of the effect of ISD on different markers tested in the same donor **(B)**. A summary of selected markers is presented in **(C)**, as % positive cells or MFIR. Data are shown as mean ± SD of 6 (for CD25, CD69 and CD54) or 3 (NKG2A and NKG2D) independent experiments. ANOVA with Dunnett's Multiple Comparison Test as post-test was used. *p-*values with statistical significance are indicated by * and † symbols for % positive cells and MFIR, respectively. *, ^†^*p* < 0.05, **, ^††^*p* < 0.01, ***, ^†††^*p* < 0.005.

The effect of ISD on NK cell phenotype was analyzed by flow cytometry and shown by surface expression levels, % of positive cells and MFIR, respectively (Figure 2 and Table S1). First, there were no differences in CD56 expression when NK cells were cultured with ISD and IVIg (data not shown). Calcineurin inhibitors (CsA and TAC); EVE; and MePRD all significantly inhibited CD69 expression; whereas CD25 expression was only inhibited by CsA and MePRD. Up-regulation of CD54 (ICAM1) expression was inhibited by all ISD. MePRD and EVE induced minor changes of the expression of the C-type lectin receptors NKG2A and NKG2D. In contrast, NCR, activating receptors DNAM-1, NKG2C, and in particular CD16 expression was not affected. IVIg had no impact on CD16 (93 ± 5 vs. 90 ± 6%, and 19 ± 12 vs. 19 ± 11 MFIR for control and IVIg, respectively), CD69 and CD25 expression at all doses tested (data not shown). Finally, no phenotype differences were observed within the NK cell sub-populations following ISD incubation (data not shown).

In summary, the effect of ISD, at concentrations corresponding to the trough levels in treated patients, on the expression of NK cell activation markers and receptors in response to IL2 was moderate, limited to CD69, CD54 and to a lesser degree to CD25, NKG2D, and NKG2A, while IVIg had no major effects.

### Partial Inhibition of NK Cell Degranulation by MePRD and Differential Effect of MPA on CD56^dim^ and CD56^bright^ NK Cell Subpopulations

**Figure 3:**
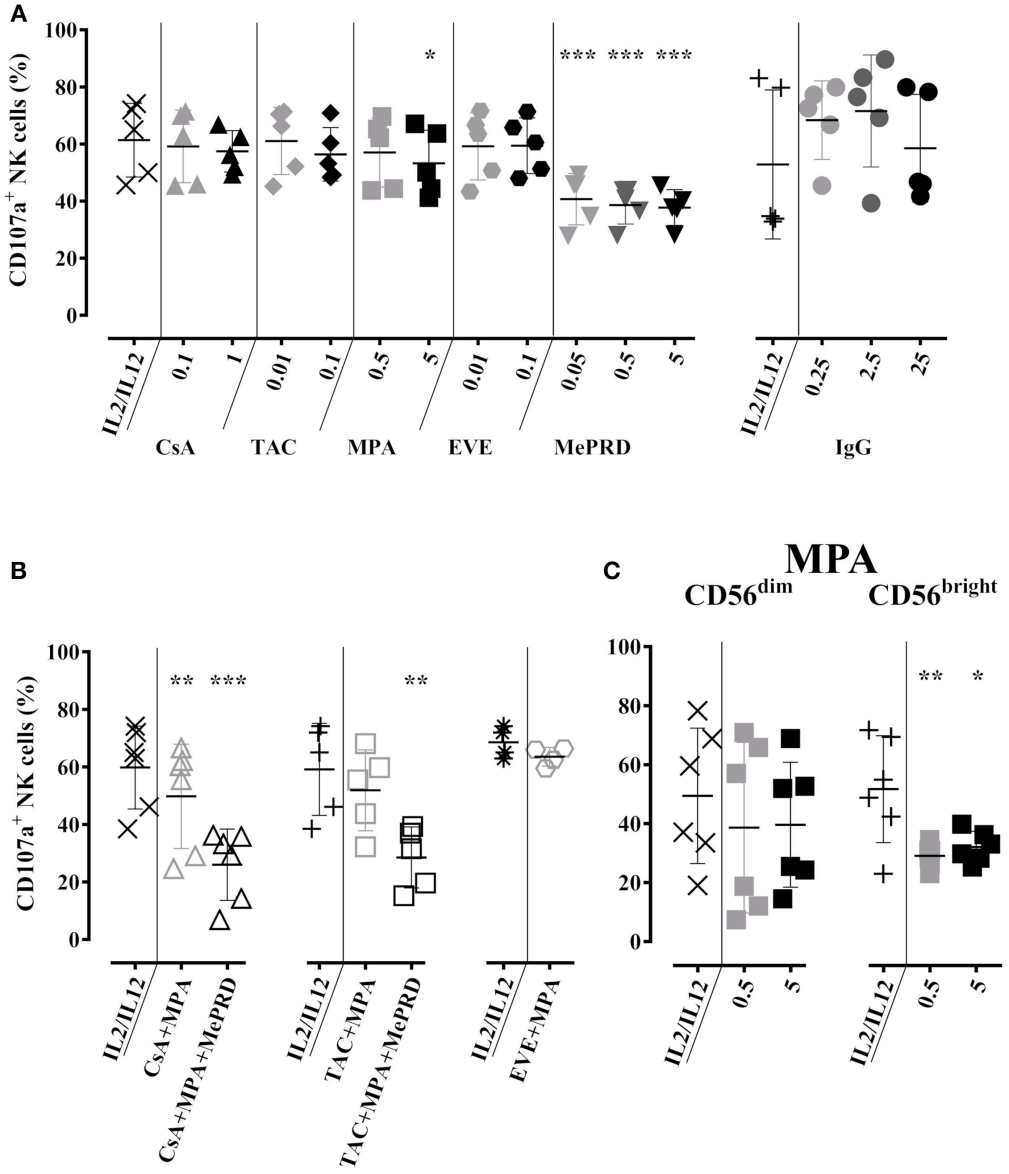
Partial inhibition of NK cell degranulation by MePRD and differential effect of MPA on CD56^dim^ and CD56^bright^ NK cell subpopulations. Purified NK cells were incubated overnight with IL2 (50 U/ml) and IL12 (0.5 ng/ml) in the presence or absence of CsA (0.1–1 μg/ml), TAC (0.01–0.1 μg/ml), MPA (0.5–5 μg/ml), EVE (0.01–0.1 μg/ml), MePRD (0.05–5 μg/ml) IVIg (0.25–25 mg/ml). Data for ISD/IVIg alone **(A)**, and ISD combinations **(B)** are shown. When used combined, the following concentrations were used: CsA (0.1 μg/ml); TAC (0.01 μg/ml); MPA (5 μg/ml); EVE (0.01 μg/ml), and MePRD (0.5 μg/ml). The specific effect of MPA on CD56^dim^ and CD56^bright^ NK subpopulations is shown in **(C)**. Carrier solutions containing ethanol (EtOH) or vehicle in the case of IVIg were used as internal controls (not shown). Degranulation was measured by detecting CD107a up-regulation on the surface of NK cells upon incubation with K562 cells for 3h at an effector to stimulator (E:S) ratio of 1:1. Golgistop was added 2h prior the end of the incubation, followed by CD56 staining and flow cytometry analysis. Data are presented as percentage of CD56^+^CD107^+^ cells ± SD. Symbols represent individual experiments, **(A)** *n* = 5 for ISD, *n* = 4 for IVIG; **(B)** *n* = 5; and **(C)** *n* = 6. Statistical analysis was performed using one-way ANOVA for matched data with Dunnett's Multiple Comparison Test as post-test vs. IL2/IL12 with the exception of EVE/MPA **(B)** were paired *t*-test was performed.*p < 0.05, **p < 0.01, ***p < 0.005.

The effect of ISD on NK cell degranulation was tested by cell surface expression of CD107a. Compared to the baseline CD107a expression following overnight incubation with IL2/IL12 and 3 h co-culture with K562 cells, a significant reduction of CD107a was observed upon treatment with MPA and MePRD (Figure 3A, left panel). Cumulative inhibition of degranulation was observed when MePRD was used in combination with CsA/MPA, or TAC/MPA, but not for the combination of EVE/MPA (Figure 3B). NK cells are grouped into two subpopulations: CD56^dim^, holding mainly cytotoxic effector functions; and CD56^bright^, essentially producing cytokines, depending on the triggering factors (34, 35). MPA had only a minor effect on degranulation of the total NK population, but specifically and strongly inhibited degranulation of CD56^bright^ NK cells (Figure 3C). This difference between CD56^bright^ and CD56^dim^ NK cells was not explained by differences in viability after culturing (data not shown). Finally, incubation with CsA, TAC, EVE (Figure 3A, left panel), and IVIg (Figure 3A, right panel) had no effect on NK cell degranulation at any dose tested. A representative dot-plot analysis of CD107a and intracellular IFNγ staining is provided in Figure S2.

### MePRD Inhibits Direct NK Cytotoxicity

**Figure 4:**
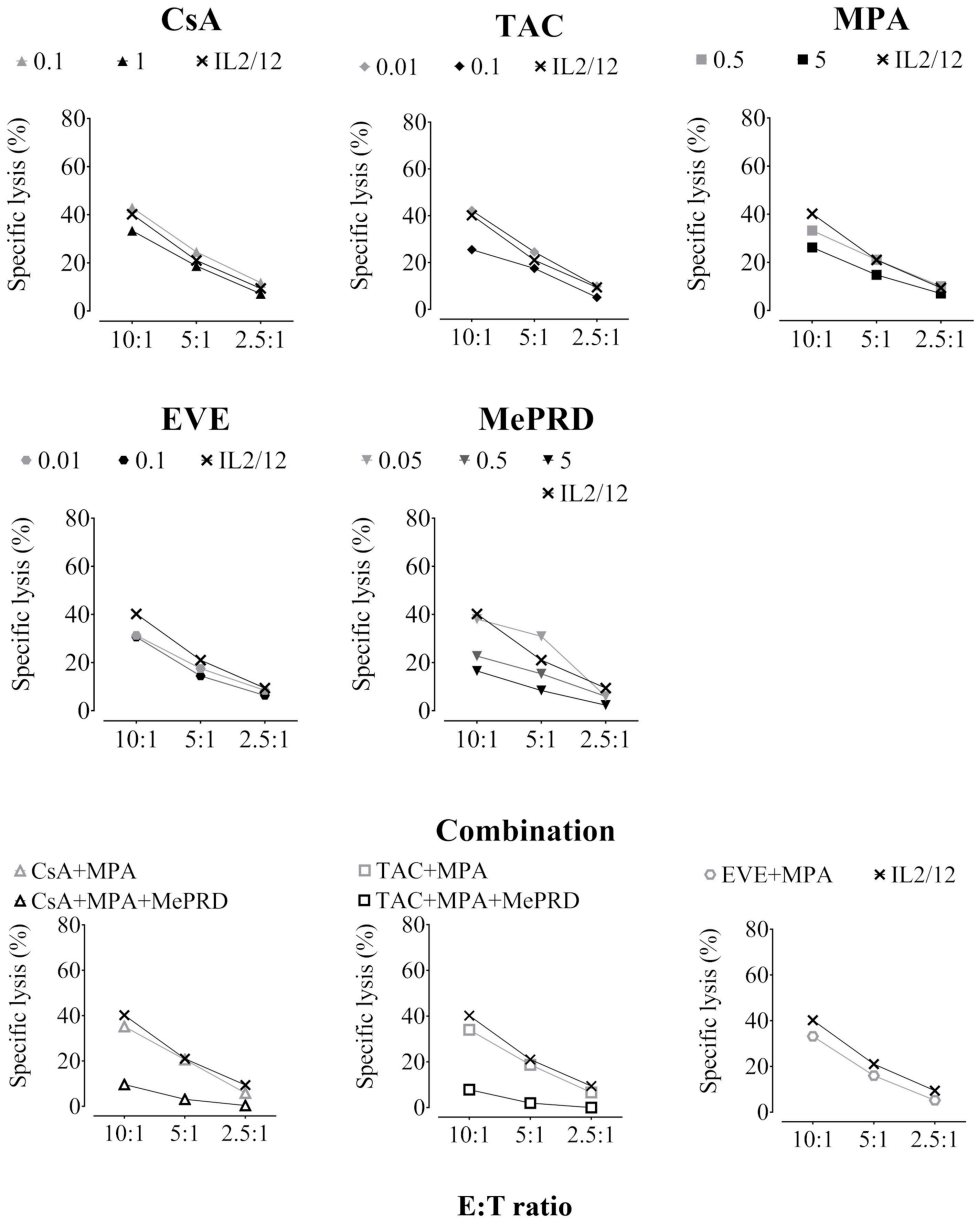
ISD inhibit direct NK cytotoxicity. Purified human NK cells were incubated overnight with IL2 (50 U/ml) and IL12 (0.5 ng/ml) in the presence or absence of CsA (0.1 and 1 μg/ml), TAC (0.01 and 0.1 μg/ml), MPA (0.5 and 5 μg/ml), EVE (0.01 and 0.1 μg/ml), MePRD (0.05–5 μg/ml) alone or in combinations without washing. When used combined, the following concentrations were used: CsA (0.1 μg/ml); TAC (0.01 μg/ml); MPA (5 μg/ml); EVE (0.01 μg/ml), and MePRD (0.5 μg/ml). Carrier solutions containing ethanol (EtOH) were used as internal controls (data not shown). NK cytotoxicity was tested using standard 4 h ^51^[Cr]-release assays. NK cells and ^51^[Cr]-labeled K562 targets were plated at different E:T ratios (10:1, 5:1, and 2.5:1). For the number of independent experiments see Table 1. Representative plots from one experiment using cells from a single donor are shown.

The effect of ISD on direct NK cytotoxicity was assessed by measuring lysis of the MHC class I-deficient NK target cell line K562. Overnight incubation with the highest concentrations of CsA, TAC, MPA and EVE resulted in a significant reduction of approximately 30% of specific lysis (Figure 4, showing representative experiments; Table 1, showing pooled data of several independent experiments). All drug combinations decreased direct NK cytotoxicity with TAC/MPA showing the strongest effect. However, MePRD, alone or in combination, was the most potent inhibitor of direct NK cytotoxicity (Table 1).

**Table 1:** Percent inhibition of direct NK cytotoxicity and ADCC by ISD.

**Pharmacological agent or combination**	**Concentration (μg/ml)**	**Direct cytotoxicity (% inhibition)**			**ADCC (% inhibition)**		
			***n***	***p***		***n***	***p***
CsA	0.1	4.2 ± 4.8	6	-	9.4 ± 6.4	7	-
	1	29.2 ± 10.7	6	^***^	ND		
TAC	0.01	10.5 ± 5.8	5	-	21.2 ± 3.1	9	^**^
	0.1	33.9 ± 12.5	5	^***^	ND		
MPA	0.5	12.9 ± 5.7	5	-	19.6 ± 11.9	9	^**^
	5	30.1 ± 7.9	5	^***^	ND		
EVE	0.01	13.9 ± 4.0	6	-	22.7 ± 4	10	^***^
	0.1	30.3 ± 10.1	6	^***^	ND		
MePRD	0.05	32.2 ± 1.7	4	^***^	52.4 ± 0.9	9	^***^
	0.5	45.1 ± 14.3	6	^***^	ND		
	5	63.3 ± 12.5	5	^***^	ND		
CsA+MPA	0.1+5	25.8 ± 6.9	4	^***^	ND		
CsA+MPA+MePRD	0.1+5+0.5	78.2 ± 9.2	4	^***^	ND		
TAC+MPA	0.01+5	40.3 ± 5.8	4	^***^	ND		
TAC+MPA+MePRD	0.01+5+0.5	86.6 ± 7.7	4	^***^	ND		
EVE+MPA	0.01+5	32.0 ± 4.2	4	^***^	ND		

Purified NK cells were incubated overnight prior to direct cytotoxicity and ADCC assays. The inhibition of cytotoxicity by pre-incubation with ISD was determined by pooling data of all E:T ratios. Cytotoxicity in the absence of ISD was used as a reference. Data are presented as percentage of inhibition ± SD and were analyzed by one-way ANOVA for matched data. The mean values of three different E:T ratios were used for the calculations, followed by Dunnett's multiple comparisons using either IL2/IL12 or sera conditions as reference for direct NK cytotoxicity or ADCC, respectively. ^*^ p < 0.05,

** *p < 0.01*,

*** *p < 0.005. ND, not determined. The number (n) of independent experiments is indicated in the table*.

### Immunosuppressive Drugs Inhibit ADCC Mediated by NK Cells

**Figure 5:**
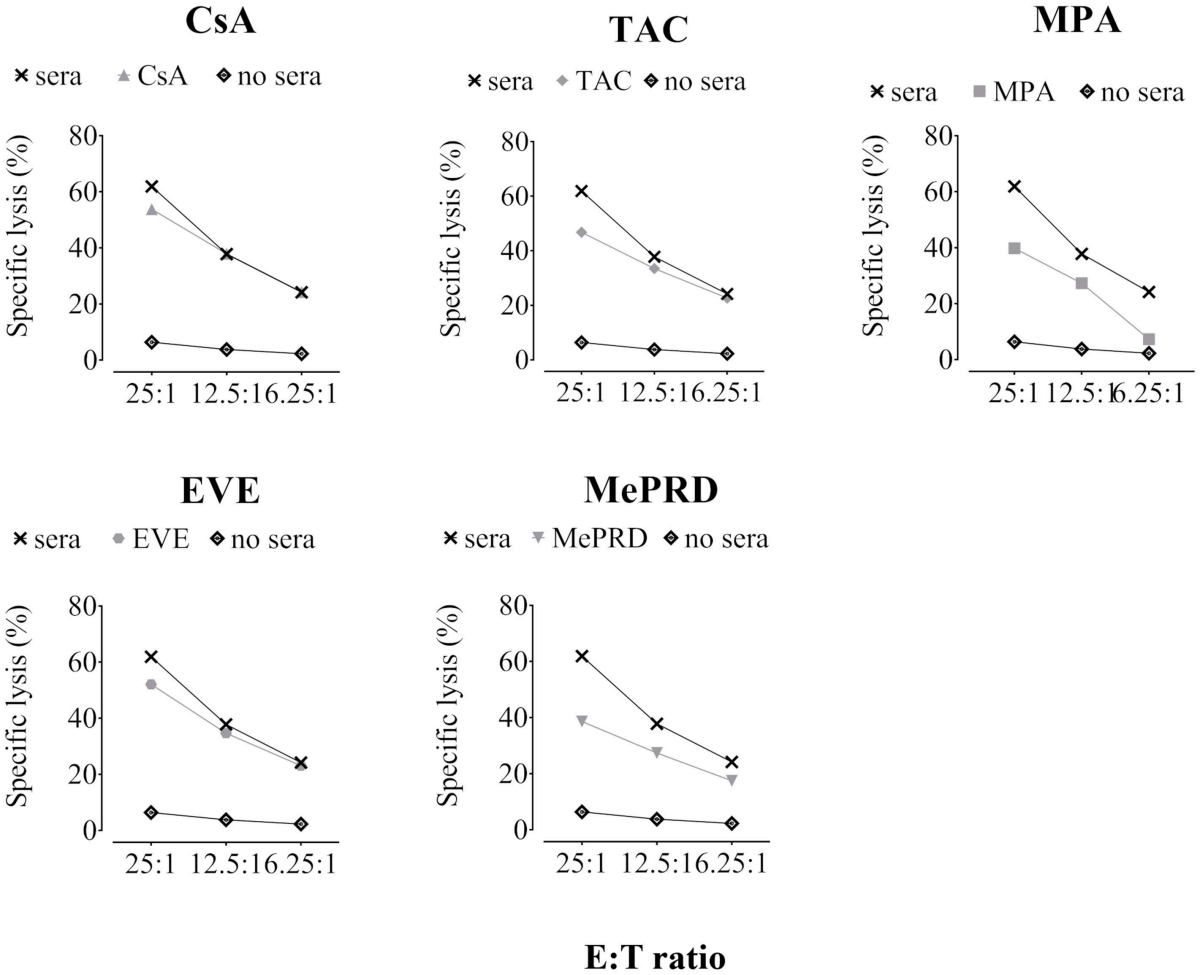
ADCC mediated by NK cells is inhibited by immunosuppressive drugs. Representative plots of ADCC from a single donor tested for different ISD are shown. Purified NK cells were incubated overnight with IL2 (50 U/ml) and IL12 (0.5 ng/ml) in the presence of CsA (0.1 μg/ml), TAC (0.01 μg/ml), MPA (5 μg/ml), EVE (0.01 μg/ml), and MePRD (0.5 μg/ml). NK effector cells and ^51^[Cr]-labeled PED target cells (pre-incubated or not for 45 min with 10% human serum) were plated at different E:T ratios (25:1, 12.5:1, and 6.25:1). After 4 h, ^51^[Cr]-release was measured. For the number of independent experiments see Table 1.

To investigate the effect of ISD on ADCC, lysis of PED was tested after incubation with human serum containing natural antibodies directed against xenoantigens expressed on the surface of porcine cells, mainly carbohydrates such as αGal1-3Gal (6). Exposure of NK cells to TAC, MPA and EVE induced a significant reduction of ADCC, whereas treatment with CsA had no significant effect (Figure 5; Table 1). MePRD demonstrated the strongest inhibition at all E:T ratios tested.

### Immunoglobulins Inhibit Direct NK Cytotoxicity and ADCC

**Figure 6:**
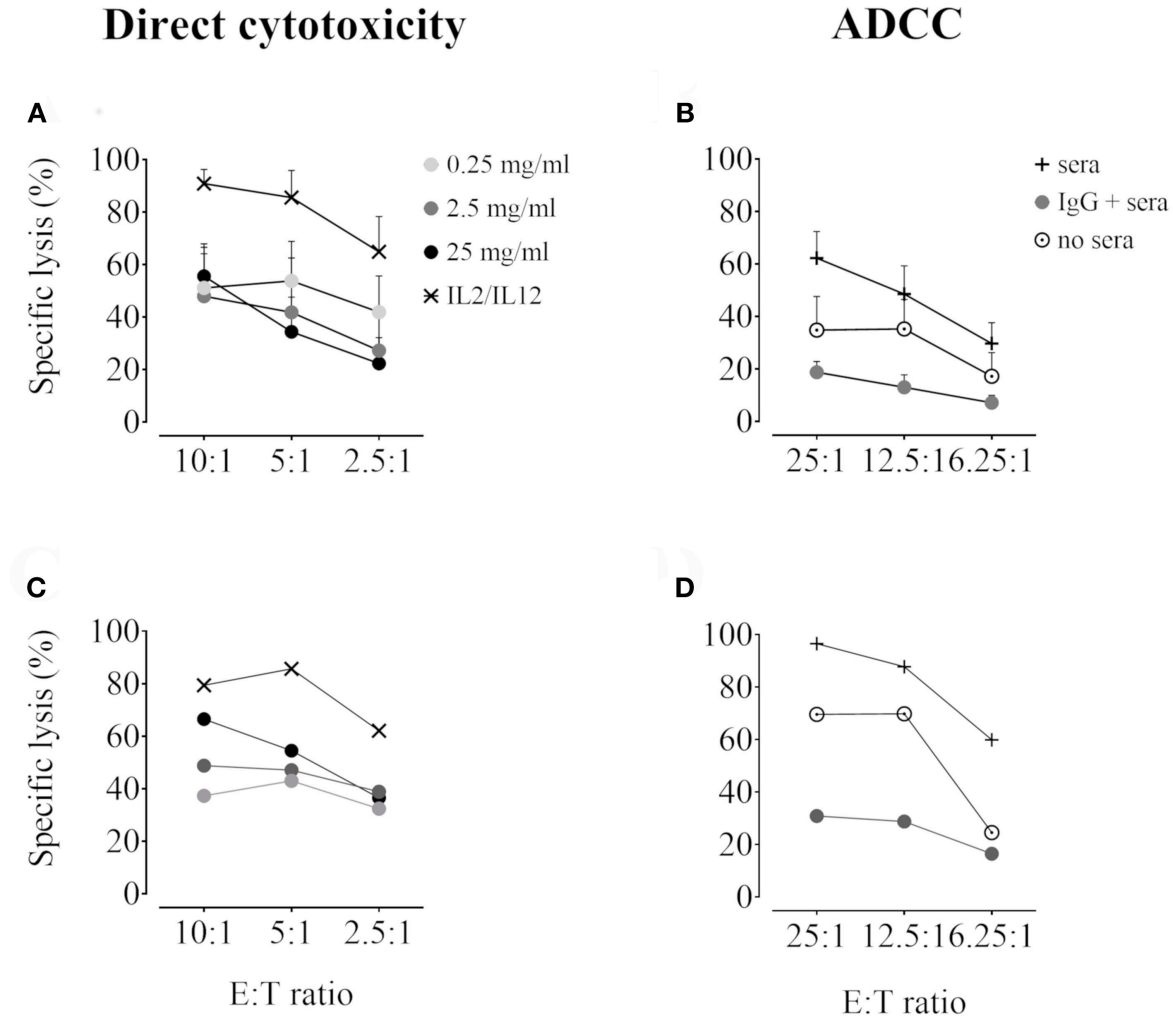
Inhibition of direct NK cytotoxicity and ADCC by IVIg. Purified human NK cells were incubated overnight with IL2 (50 U/ml) and IL12 (0.5 ng/ml) in the presence or not of IVIg (0.25–25 mg/ml). Vehicle was used as an internal control (data not shown). NK cytotoxicity was tested using DELFIA cytotoxicity assays. **(A)** and **(C)** Direct NK cytotoxicity: NK cells and BATDA-labeled K562 targets were plated at different E:T ratios (10:1, 5:1, and 2.5:1) for 2 h. **(B,D)** ADCC: BATDA-labeled PED target cells (pre-incubated or not for 45 min with 10% human serum) were plated at different E:T ratios (25:1, 12.5:1, and 6.25:1) for 2 h with IVIg (2.5 mg/ml). In both assays, EuTDA-release was measured by time-resolved fluorometry. Data are presented as specific lysis plots, in **(A,B)** as pooled data (*n* = 6 for direct cytotoxicity, *n* = 5 for ADCC), and in **(C,D)** as a single donor representative experiment.

IVIg preincubation decreased direct NK lysis of K562 cells at all doses tested (Figure 6A, pooled data and Figure 6C representative experiment), with the most potent effect at 2,5 and 25 mg/ml (53 ± 8.5% and 52.7 ± 12.5% overall inhibition in 5 independent pooled experiments, respectively). Moreover, exposure to IVIg resulted in virtually complete inhibition of ADCC in addition to partial blocking of direct NK cytotoxicity against porcine endothelial cells (Figure 6B, pooled data and Figure 6D representative experiment). The overall inhibition of ADCC in five independent experiments was 95.5 ± 10.6%.

### Inhibition of NK Cell IFNγ Production and Secretion by MePRD and a Combination of Calcineurin Inhibitors and MPA

**Figure 7:**
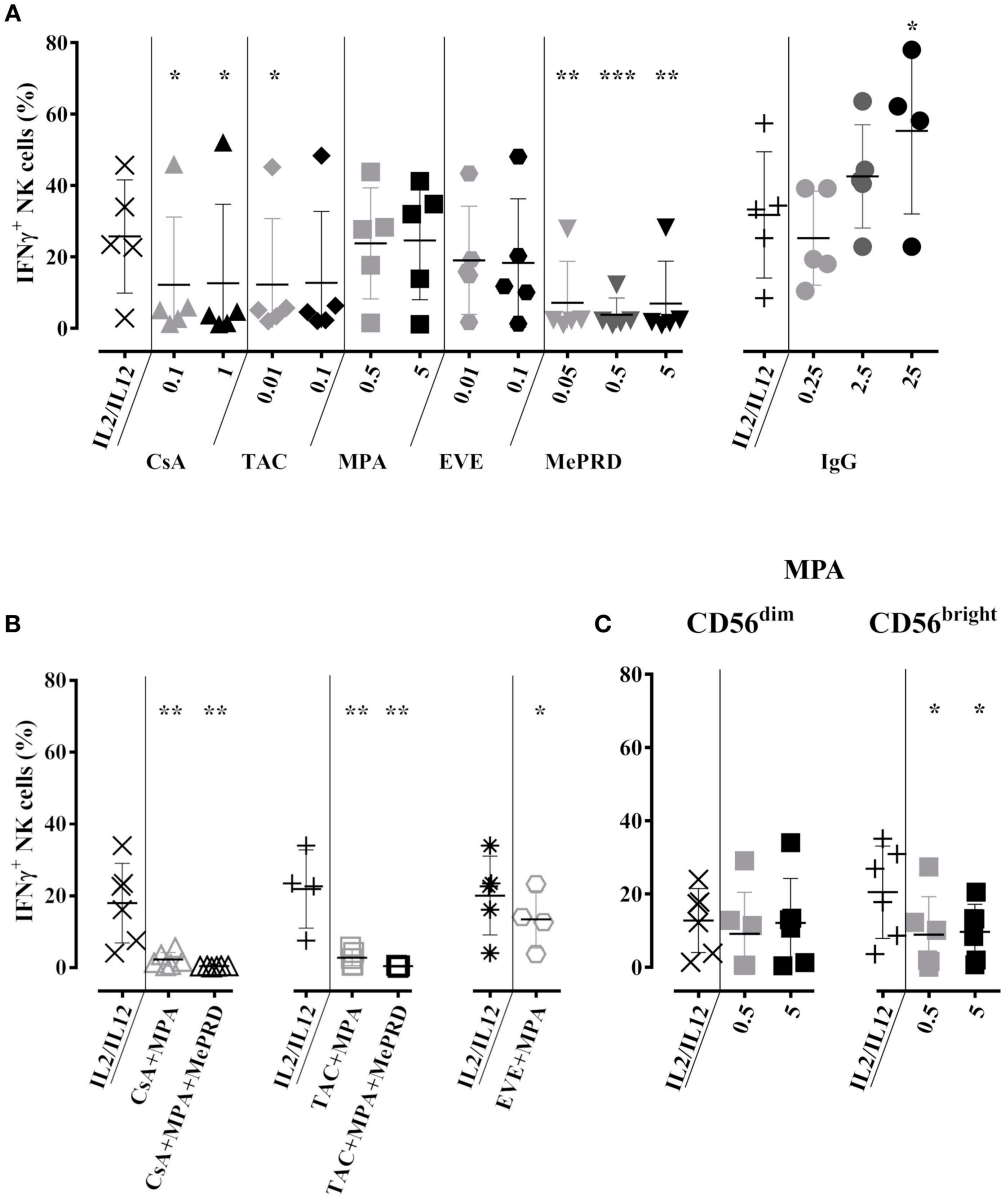
Inhibition of NK cell IFNγ production by MePRD and calcineurin inhibitors but not by IVIg. Purified NK cells were incubated overnight with IL2 (50 U/ml) and IL12 (0.5 ng/ml) in the presence or absence of CsA (0.1–1 μg/ml), TAC (0.01–0.1 μg/ml), MPA (0.5–5 μg/ml), EVE (0.01–0.1 μg/ml), MePRD (0.05–5 μg/ml) IVIg (2,5–25 mg/ml) **(A)** alone or **(B)** in combination, while **(C)** shows the specific effect of MPA on CD56^dim^ and CD56^high^ NK subpopulations. When used combined, the following concentrations were used: CsA (0.1 μg/ml); TAC (0.01 μg/ml); MPA (5 μg/ml); EVE (0.01 μg/ml), and MePRD (0.5 μg/ml). IL2 and IL12 only treated cells were used as control. Carrier solution containing either EtOH or vehicle in the case of IVIg was used as an internal control (data not shown). IFNγ production was detected by intracellular flow cytometry analysis upon additional incubation with K562 stimulator cells for 3 h at an E:S ratio of 1:1. After two hours of incubation, Golgistop was added followed by CD56 staining and flow cytometry analysis. Data are presented as mean percentage of CD56^+^IFNγ^+^ cells ± SD. Symbols represent individual experiments, **(A)** *n* = 5 or 6 for ISD; *n* = 5 for IVIg; **(B)**, *n* = 5; and **(C)** *n* = 6. Statistical analysis was performed using one-way ANOVA for matched data with Dunnett's Multiple Comparison Test as post-test *vs*. IL2/IL12 with the exception of EVE/MPA **(B)** were paired *t*-test was performed. *p < 0.05, **p < 0.01, ***p < 0.005.

The effect of ISD and IVIg on IFNγ production was first assessed by intracellular IFNγ staining after overnight stimulation with IL2/IL12 and 3 h of K562 target cell co-culture. Both doses of CsA and the low dose of TAC partially inhibited IFNγ production by approximately 50%. Overall, MePRD was the most potent inhibitor, whereas MPA and EVE had no significant effect (Figure 7A, left panel). It is of note that one particular donor exhibited very high levels of intracellular IFNγ that did not respond to the treatment by ISD. In contrast, higher doses of IVIg rather increased IFNγ production (Figure 7A, right panel). Almost complete inhibition of IFNγ production was observed when ISD were used in combinations, with the exception of EVE/MPA (Figure 7B). When MPA was separately tested in the CD56^dim^ and CD56^bright^ NK subpopulations, CD56^dim^ NK cells were relatively resistant, whereas CD56^bright^ NK cells were specifically inhibited by both doses of MPA (Figure 7C).

**Figure 8:**
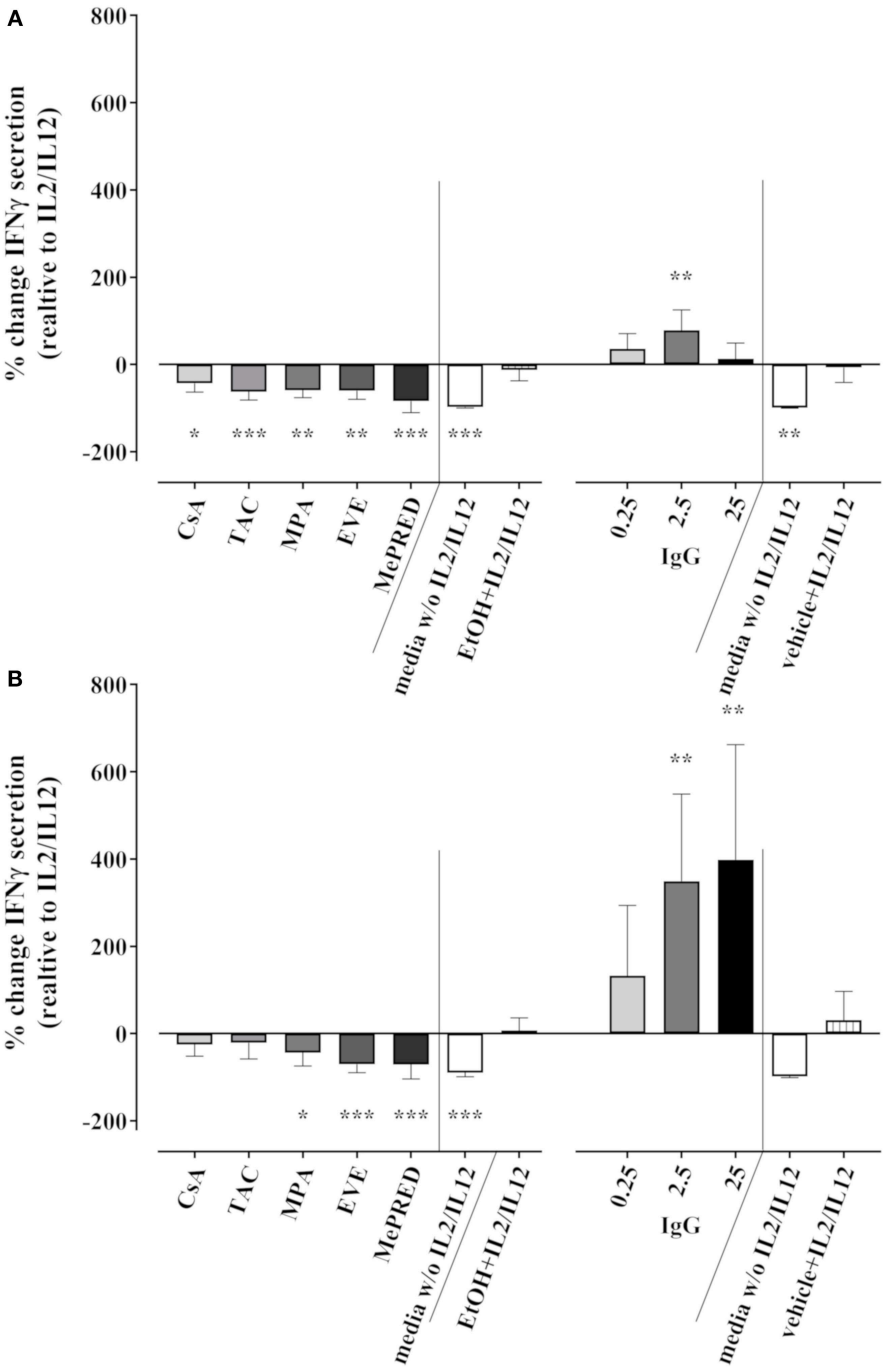
Effect of ISD and IVIg on IFNγ secretion by NK cells in response to IL2 and IL12. Secretion of IFNγ after overnight incubation of purified NK cells with IL2 (50 U/ml) and IL12 (0.5 ng/ml) in the presence or absence of CsA (0.1 μg/ml), TAC (0.01 μg/ml), MPA (5 μg/ml), EVE (0.01 μg/ml), MePRD (0.5 μg/ml), and IVIg (0.25–25 mg/ml) was measured in the culture supernatants by ELISA (limit of detection was 15.6 pg/ml); **(A)** upon additional incubation with K562 stimulator cells for 3 h at an E:S ratio of 1:1; **(B)** with no additional stimulation by K562. Combinations of ISD were not analyzed. Percentage of change of IFNγ secretion induced by ISD compared to IL2/IL12 alone is shown. Controls included NK cell cultures in medium without IL2/IL12 or in EtOH/vehicle with IL2/IL12. Statistical analysis was performed using one-way ANOVA for matched data with Dunnett's Multiple Comparison Test as post-test vs. IL2/IL12. Data are presented as percentage of control ± SD. (*n* = 3 drugs alone, *n* = 5 for IVIg).

We also tested the effect of ISD/IVIg on IFNγ secretion into culture supernatants by ELISA following 18 h of cytokine stimulation and 3 h K562 co-culture. Taking the absolute IFNγ concentrations into account, the secretion of IFNγ into stimulated NK cell culture supernatants was not significantly inhibited by ISD with the exception of MePRD. Compared to the control (1292 ± 999 pg/ml), 952 ± 826, 227 ± 140, 826 ± 827 and 780 ± 742 pg/ml were measured for CsA, TAC, MPA and EVE, respectively (data not shown); while MePRD decreased IFNγ secretion (20 ± 19 pg/ml). However, considerable inter-donor variations in IFNγ secretion and probably the rather short overall duration of stimulation rendered the interpretation of pooled data unreliable. Therefore, the relative change of IFNγ secretion induced by ISD/IVIg was calculated for each NK cell donor (*n* = 3) independently. As shown in Figure 8, TAC and MePRD reduced the secretion of IFNγ significantly by 71.1 ± 24.5% and 95.6 ± 5.5%, respectively; whereas MPA and EVE by 42.4 ± 19.8% and 42.9 ± 15.3%, respectively, CsA-induced changes were barely significant (27.3 ± 12.16%) (Figure 8A, left panel). Equivalent results were found when NK cells were stimulated only with cytokines in the absence of target cells (Figure 8B, left panel). Overnight treatment of NK cells with IVIg had no significant effect on IFNγ secretion (Figure 8A, right panel) with the exception of 2.5 mg/ml that increased the secretion. On the other hand, when IVIg was added on NK cell cultures containing only cytokines, a dose-dependent increase in IFNγ secretion was observed (Figure 8B, right panel).

In summary, intracellular IFNγ production, as well as IFNγ secretion was inhibited by ISD with MePRD showing the strongest effect. In contrast, IVIg showed a trend toward increased IFNγ production and secretion.

## Discussion

Several studies have examined the effect of ISD on NK cells obtained from healthy donors *in vitro* (36–45) and a few studies *ex vivo*, using blood samples from immunosuppressed patients (46–51). However, the current evidence has never been consolidated and remains somewhat inconclusive, mostly due to different experimental designs (Table 2). Other studies performed with rodent NK cells, the NK92 cell line, very high cytokine stimulation (1,000 U/ml IL2), or some older reports which were performed using PBMC rather than purified NK cells to assess cytotoxicity are hard to compare (59–62).

**Table 2:** Effect of immunosuppressive drugs and IVIg on NK cell functions *in vitro*.

**Drug**	**Proliferation of purified NK cells (or PBMC)**	**Stimulation**			**Method**	**References**
		**Stimuli**	**Dose**	**Duration**		
MePRD PRED DEX	**Inhibition**	**IL2**	**50 U/ml**	**5d**	**^3^[H] incorporation**	**Current study**
	Inhibition	IL2, IL15	20–200 U/ml, 5–50 ng/ml	5d	Cell counting, trypan blue	(36)
	Inhibition (PBMC)	TEC	–	7d	FACS	(37)
	Inhibition	IL2	100 U/ml	7d	FACS	(43)
CsA	**Inhibition**	**IL2**	**50 U/ml**	**5d**	**^3^[H] incorporation**	**Current study**
	Inhibition (PBMC)	γ-irr. K562	–	2d	FACS	(46)
	Inhibition	IL2/IL15	100 U/ml + 10 U/ml	7d	FACS	(41)
	Inhibition	IL2	100 U/ml	7d	FACS	(43)
	Inhibition	IL2 + IL15	100 U/ml + 10 ng/ml	1, 3, 5, 9d	FACS	(42)
	Inhibition	IL2 + IL15	100 U/ml + 10 ng/ml	5d	FACS	(38)
	No effect (PBMC)	γ-irr. K56	–	4d	FACS	(46)
TAC	**Inhibition**	**IL2**	**50 U/ml**	**5d**	**^3^[H] incorporation**	**Current study**
	Inhibition (PBMC)	γ-irr. K562	–	2d	FACS	(46)
	Inhibition (PBMC)	TEC	–	7d	FACS	(37)
	Inhibition	IL2	100 U/ml	7d	FACS	(43)
	Inhibition	IL2 + IL15	100 U/ml + 10 ng/ml	1, 3, 5, 9d	FACS	(42)
	Inhibition	IL2	300 U/ml	7d	^3^[H] incorporation	(45)
	No effect (PBMC)	γ-irr. K562	–	4d	FACS	(46)
EVE RAPA	**Inhibition**	**IL2**	**50 U/ml**	**5d**	**^3^[H] incorporation**	**Current study**
	Inhibition (PBMC)	TEC	–	7d	FACS	(37)
	Inhibition	IL2 + IL15	100 U/ml + 10 ng/ml	5d	FACS	(38)
	Inhibition	IL2	100 U/ml	7d	FACS	(43)
MMF MPA	**Inhibition**	**IL2**	**50 U/ml**	**5d**	**^3^[H] incorporation**	**Current study**
	Inhibition (PBMC)	γ-irr. K562	–	2d	FACS	(46)
	Inhibition	IL2	100 U/ml	7d	FACS	(43)
	Inhibition	IL2 + IL15	100 U/ml + 10 ng/ml	1, 3, 5, 9d	FACS	(42)
	Inhibition	IL2 + IL15	100 U/ml + 10 ng/ml	5d	FACS	(38)
	Inhibition	IL2	1,000 U/ml	9d	FACS	(52)
	No Effect (PBMC)	γ-irr. K562	–	4d	FACS	(46)
IVIg	**No effect**	**IL2**	**50 U/ml**	**5d**	**^3^[H] incorporation**	**Current study**
	Increase	IL2	6,000 U/ml	3d	^3^[H] incorporation	(44)
**Drug**	**Direct cytotoxicity of purified NK cells (or PBL)**	**Stimulation**			Method (time, h*)	**References**
		**Pre-stimulation**	**Dose (duration)**	**Target cells**		
MePRD PRED DEX	**Inhibition**	**IL2** **+** **IL12**	**50 U/ml** **+** **0.5 ng/ml (18 h)**	**K562**	^**51**^**[Cr]-release (4 h)**	**Current study**
	Inhibition	–	–	K562	^51^[Cr]-release (4 h)	(48)
	Inhibition	IL2 + IL12 + 721.221	250 U/ml + 10 U/ml (3d)	721.221	FACS	(48)
	Inhibition	IL2, IL15	100 U/ml, 20 ng/ml (5d)	721.221, FO1	^51^[Cr]-release (4 h)	(36)
	Inhibition	IL2	100 U/ml (5d)	iDC, CHO, FO1, M14, 721.221	^51^[Cr]-release (4 h)	(40)
	Inhibition	**–**	**–**	TEC	TDA-release (4 h)	(37)
	Inhibition	–	–	K562	FACS (1 h)	(43)
	Inhibition	IL2 + IL12 + 721.221	– (3d)	721.221	FACS (3 h)	(48)
	Inhibition (PBL)	–	–	K562	FACS (2 h)	(39)
CsA	**Inhibition**	**IL2** **+** **IL12**	**50 U/ml** **+** **0.5 ng/ml (18 h)**	**K562**	^**51**^**[Cr]-release (4 h)**	**Current study**
	Inhibition	IL2 + IL12 + 721.221	250 U/ml + 10 U/ml (3d)	721.221	FACS	(48)
	No effect	**–**	**–**	K562	^51^[Cr]-release (4 h)	(48)
	No effect	IL2 + IL15	100 U/ml + 10 ng/ml (5d)	K562	^51^[Cr]-release (4 h)	(38)
	No effect	IL2 + IL15	100 U/ml + 10 ng/ml (7d)	K562, DAUDI	^51^[Cr]-release	(42)
	No effect	**–**	**–**	K562	FACS (1 h)	(43)
	Augmentation	IL2 + IL15	100 U/ml + 10 U/ml (7d)	K562, Raji, LCL	^51^[Cr]-release (4 h)	(41)
EVE RAPA	**Inhibition**	**IL2** **+** **IL12**	**50 U/ml** **+** **0.5 ng/ml (18 h)**	**K562**	^**51**^**[Cr]-release (4 h)**	**Current study**
	Inhibition	IL2 + IL15	100 U/ml + 10 ng/ml (5d)	K562	^51^[Cr]-release (4 h)	(38)
	No effect	–	–	TEC	TDA-release (4 h)	(37)
	No effect	**–**	**–**	K562	FACS (1 h)	(43)
	No effect	IL2 + IL15	100 U/ml + 10 ng/ml (1d, 7d)	MSC	TDA-release (4 h)	(53)
TAC	**Inhibition**	**IL2** **+** **IL12**	**50 U/ml** **+** **0.5 ng/ml (18 h)**	**K562**	^**51**^**[Cr]-release (4 h)**	**Current study**
	Inhibition	IL2	300 U/ml (7d)	K562, CEM, U937	^51^[Cr]-release (4 h)	(45)
	No effect	IL2 + IL15	100 U/ml + 10 ng/ml (7d)	K562, DAUDI,	^51^[Cr]-release	(42)
	No effect	**–**	**–**	K562	FACS (1 h)	(43)
	No effect	IL2 + IL15	100 U/ml + 10 ng/ml (1d, 7d)	MSC	TDA-release (4 h)	(53)
	No effect	**–**	**–**	TEC	TDA-release (4 h)	(37)
MMF MPA	**Inhibition**	**IL2** **+** **IL12**	**50 U/ml** **+** **0.5 ng/ml (18 h)**	**K562**	^**51**^**[Cr]-release (4 h)**	**Current study**
	Inhibition	**–**	**–**	K562	^51^[Cr]-release (4 h)	(48)
	Inhibition	IL2 + IL15	100 U/ml + 10 ng/ml (5d)	K562	^51^[Cr]-release (4 h)	(38)
	Inhibition	IL2 + IL15	100 U/ml + 10 ng/ml (7d)	K562, DAUDI	^51^[Cr]-release	(42)
	Inhibition	IL2	1,000 U/ml (9, 10d)	K562	FACS	(52)
	Inhibition	IL2 + IL12 + 721.221	250 U/ml + 10 U/ml (3d)	721.221	FACS	(48)
	No effect	**–**	**–**	K562	FACS (1 h)	(43)
IVIg	**Inhibition**	**IL2** **+** **IL12**	**50 U/ml** **+** **0.5 ng/ml (18 h)**	**K562**	**TDA-release (2 h)**	**Current study**
	Inhibition	IL2	6,000 U/ml (2, 3d)	K562, DAUDI	^51^[Cr]-release (4 h)	(44)
	Inhibition	(PBL) –	–	K562	FACS (2 h)	(39)
	Augmentation	**–**	**–**	K562, DAUDI	^51^[Cr]-release (4 h)	(44)
**Drug**	Degranulation of purified NK cells (*or PBMC) by CD107a FACS	**Stimulation**			**Assay duration**	**References**
		**Pre-stimulation**	**Dose (duration)**	**Targets**		
MePRD PRED DEX	**Inhibition**	**IL2** **+** **IL12**	**50 U/ml** **+** **0.5 ng/ml (18 h)**	**K562**	**4 h**	**Current study**
	Inhibition (PBMC)	–	–	K562	6 h	(48)
	No effect (PBMC)	TEC	– (7d)	Sorted NK cells, TEC	2 h	(37)
CsA	**No effect**	**IL2** **+** **IL12**	**50 U/ml** **+** **0.5 ng/ml (18 h)**	**K562**	**4 h**	**Current study**
	Inhibition (PBMC)	IL2	100 U/ml (18 h)	K562	6 h	(49)
	No effect (PBMC)	–	–	K562	6 h	(48)
TAC	**No effect**	**IL2** **+** **IL12**	**50 U/ml** **+** **0.5 ng/ml (18 h)**	**K562**	**4 h**	**Current study**
	Inhibition	IL2	300 U/ml (7d)	K562	6 h	(45)
	Inhibition (PBMC)	IL2	100 U/ml (18 h)	K562	6 h	(49)
	Inhibition (PBMC)	TEC	– (7d)	Sorted NK cells, TEC	2 h	(37)
MMF MPA	**Inhibition**	**IL2** **+** **IL12**	**50 U/ml** **+** **0.5 ng/ml (18 h)**	**K562**	**4 h**	**Current study**
	Inhibition (PBMC)	–	–	K562	6 h	(48)
EVE	**No effect**	**IL2** **+** **IL12**	**50 U/ml** **+** **0.5 ng/ml (18 h)**	**K562**	**4 h**	**Current study**
	No effect (PBMC)	TEC	– (7d)	Sorted NK cells, TEC	2 h	(37)
IVIg	**No effect**	**IL2** **+** **IL12**	**(18 h)**	**K562**	**4 h**	**Current study**
	Augmentation (whole blood)		–	K562	3 h	(54)
MePRD PRED DEX	**Inhibition**	**IL2** **+** **IL12**	**50 U/ml** **+** **0.5 ng/ml (18 h)**	**Ab-coated PED**	^**51**^**[Cr]-release (4 h)**	**Current study**
	Inhibition (PBMC)	–	–	Rituximab + CD20^+^ lymphoma	^51^[Cr]-release (4 h)	(55)
	Inhibition (whole blood)	–	–	_γ−*irr*._PBMC + reactive hu Abs	CD107a FACS (18 h)	(56)
	Inhibition	–	–	Cetuximab + EGF^+^ carcinoma	LHD assay	(57)
	Inhibition (PBMC)	–	–	Cetuximab + EGF^+^ carcinoma	LHD assay	(57)
	Inhibition, rev.ADCC	IL2	100 U/ml (5d)	α-CD16 + P815	^51^[Cr]-release (4 h)	(40)
	No effect, rev.ADCC	IL2	100 U/ml (5d)	α-CD16 + P815	^51^[Cr]-release (4 h)	(36)
CsA	**No effect**	**IL2** **+** **IL12**	**50 U/ml** **+** **0.5 ng/ml (18 h)**	**Ab-coated PED**	^**51**^**[Cr]-release (4 h)**	**Current study**
	Inhibition (PBMC)	IL2	500 U/ml (2d)	α-CD16 plate-bound	CD107a FACS (4 h)	(46)
	Inhibition (whole blood)	–	–	_γ−*irr*._PBMC + reactive hu Abs	CD107a FACS (18 h)	(56)
TAC	**Inhibition**	**IL2** **+** **IL12**	**50 U/ml** **+** **0.5 ng/ml (18 h)**	**Ab-coated PED**	^**51**^**[Cr]-release (4 h)**	**Current study**
	Inhibition (whole blood)	–	–	_γ−*irr*._PBMC + reactive hu Abs	CD107a FACS (18 h)	(56)
	Inhibition (PBMC)	IL2	500 U/ml (2d)	α-CD16 plate-bound	CD107a FACS (4 h)	(46)
MMF MPA	**Inhibition**	**IL2** **+** **IL12**	**50 U/ml** **+** **0.5 ng/ml (18 h)**	**Ab-coated PED**	^**51**^**[Cr]-release (4 h)**	**Current study**
	Inhibition (whole blood)	–	–	_γ−*irr*._PBMC + reactive hu Abs	CD107a FACS (18 h)	(56)
	No effect (PBMC)	IL2	500 U/ml (2d)	α-CD16 plate-bound	CD107a FACS (4 h)	(46)
EVE RAPA	**Inhibition**	**IL2** **+** **IL12**	**50 U/ml** **+** **0.5 ng/ml (18 h)**	**Ab-coated PED**	^**51**^**[Cr]-release (4 h)**	**Current study**
	Inhibition (whole blood)	–	–	_γ−*irr*._PBMC + reactive hu Abs	CD107a FACS (18 h)	(56)
	Inhibition (PBMC)	IL2	500 U/ml (2d)	α-CD16 plate-bound	CD107a FACS (4 h)	(46)
CsA + PRED	Inhibition (whole blood)	–	–	_γ−*irr*._PBMC + reactive huAbs	CD107a FACS (18 h)	(56)
TAC + PRED	Inhibition (whole blood)	–	–	_γ−*irr*._PBMC + reactive hu Abs	CD107a FACS (18 h)	(56)
IVIg	**Inhibition**	**IL2** **+** **IL12**	**50 U/ml** **+** **0.5 ng/ml (18 h)**	**Ab-coated PED**	**TDA-release (2 h)**	**Current study**
**Drug**	**IFNγ** **production (or other cytokines) of purified NK cells (or PBMC)**	**Stimulation**			**Method**	**References**
		**Pre-stimulation**	**Dose (duration)**	**Stimuli (assay duration)**		
MePRD PRED DEX	**Inhibition**	**IL2** **+** **IL12**	**50 U/ml** **+** **0.5 ng/ml (18 h)**	**K562 (4 h)**	**FACS**	**Current study**
	Inhibition (PBMC)	**–**	**–**	K562 (6 h)	FACS	(48)
	**Inhibition**	**IL2** **+** **IL12**	**50 U/ml** **+** **0.5 ng/ml (18 h)**	**K562 (3 h)**	**ELISA**	**Current study**
	Inhibition (PBMC)	**–**	**–**	Cetuximab + EGF^+^ carcinoma	ELISA	(57)
CsA	**Inhibition**	**IL2** **+** **IL12**	**50 U/ml** **+** **0.5 ng/ml (18 h)**	**K562 (4 h)**	**FACS**	**Current study**
	Inhibition	IL2	100 U/ml (18 h)	K562 (6 h)	FACS	(49)
	Inhibition (PBMC)	**–**	**–**	K562 (6 h)	FACS	(48)
	Inhibition	PMA + IONO	50 ng/ml + 2.5 μg/ml (6d)	–	FACS	(58)
	Augmentation	IL2 + IL15	100 U/ml + 10 U/ml (7d)	IL12 + IL18 (10 +100 ng/ml, 18 h)	FACS	(41)
	**Inhibition**	**IL2** **+** **IL12**	**50 U/ml** **+** **0.5 ng/ml (18 h)**	**K562 (3 h)**	**ELISA**	**Current study**
	Inhibition	IL2 + IL15	100 U/ml + 10 ng/ml (5d)	**K562** (4 h)	ELISA	(38)
	Inhibition (TNF, IL22, IL31)	PMA + IONO	50 ng/ml + 2.5μg/ml (24 h)	– (2d)	ELISA	(58)
	Inhibition (PBMC)	–	–	K562 (18 h)	ELISPOT	(46)
TAC	**Inhibition**	**IL2** **+** **IL12**	**50 U/ml** **+** **0.5 ng/ml (18 h)**	**K562 (4 h)**	**FACS**	**Current study**
	Inhibition	IL2	100 U/ml (18 h)	K562 (6 h)	FACS	(49)
	Inhibition (TNF)	IL2	300 U/ml (7d)	K562 (6 h)	FACS	(45)
	Inhibition	PMA + IONO	50 ng/ml + 2.5 μg/ml	– (6 h)	FACS	(58)
	Inhibition (PBMC)	**–**	**–**	K562 (6 h)	FACS	(48)
	Inhibition (PBMC)	**–**	**–**	K562 (6 h)	FACS	(48)
	**Inhibition**	**IL2** **+** **IL12**	**50 U/ml** **+** **0.5 ng/ml (18 h)**	**K562 (3 h)**	**ELISA**	**Current study**
	Inhibition (TNF, IL22, IL31)	PMA + IONO	50 ng/ml + 2.5 μg/ml	– (2d)	ELISA	(58)
MMF MPA	**No effect**	**IL2** **+** **IL12**	**50 U/ml** **+** **0.5 ng/ml (18 h)**	**K562 (4 h)**	**FACS**	**Current study**
	Inhibition	PMA + IONO	50 ng/ml + 2.5 μg/ml	– (6h)	FACS	(58)
	Inhibition (PBMC)	**–**	**–**	K562 (6 h)	FACS	(48)
	**Inhibition**	**IL2** **+** **IL12**	**50 U/ml** **+** **0.5 ng/ml (18 h)**	**K562 (3 h)**	**ELISA**	**Current study**
	Inhibition	IL2 + IL15	/5d	–	ELISA	(38)
	Inhibition (TNF, IL6, IL2)	IL2	1,000 U/ml (9d)	**–**	ELISA	(52)
	No effect (TNF, IL22, IL31)	–	–	PMA + IONO (2d)	ELISA	(58)
	No effect (PBMC)	–	–	K562 (18 h)	ELISPOT	(46)
EVE RAPA	**No effect**	**IL2** **+** **IL12**	**50 U/ml** **+** **0.5 ng/ml (18 h)**	**K562 (4 h)**	**FACS**	**Current study**
	No effect	PMA + IONO	50 ng/ml + 2.5 μg/ml	– (6h)	FACS	(58)
	**Inhibition**	**IL2** **+** **IL12**	**50 U/ml** **+** **0.5 ng/ml (18 h)**	**K562 (3 h)**	**ELISA**	**Current study**
	Inhibition	IL2 + IL15	100 U/ml + 10 ng/ml (5d)	**K562** (4 h)	ELISA	(38)
	No effect (TNF, IL22, IL31)	PMA + IONO	50 ng/ml + 2.5μg/ml (24 h)	– (2d)	ELISA	(58)
	No effect (TNF, IL22, IL31)	–	–	PMA + IONO (2d)	ELISA	(58)
	No effect (PBMC)	–	–	K562 (18 h)	ELISPOT	(46)
IVIg	**Augmentation**	**IL2** **+** **IL12**	**50 U/ml** **+** **0.5 ng/ml (18 h)**	**K562 (4 h)**	**FACS**	**Current study**
	**Augmentation**	**IL2** **+** **IL12**	**50 U/ml** **+** **0.5 ng/ml (18 h)**	**K562 (3 h)**	**ELISA**	**Current study**
	Augmentation (TNF, IL1β, sIL2R)	IL2	6,000 U/ml/ 3d	**–** (3d)	ELISA	(44)
	Augmentation (whole blood)	–	–	**–**	ELISA	(54)

3 *[H], thymidine tritium incorporation; Ab, antibody; ADCC, antibody-dependent cellular cytotoxicity; CEM, human T lymphoblast cell line; CsA, cyclosporine A; EGF, epidermal growth factor; ELISA, enzyme-linked immunosorbent assay; ELISPOT, enzyme-linked immunospot; EVE, everolimus; hu, human; FACS, flow activated cell sorting; iDC, immature dendritic cell; IFNγ, interferon gamma; IL, interleukin; IONO, ionomycin; IVIg, polyspecific soluble immunoglobulins; γ-irr., gamma-irradiated; LHD, lactate dehydrogenase; MePRD, methylprednisolone; MMF, mycophenolate mofetil; MPA, mycophenolic acid; MSC, mesenchymal stem cells; NK, natural killer; PBL, peripheral blood lymphocytes; PBMC, peripheral blood mononuclear cells; PED, pig endothelial cells; PMA, phorbol 12-myristate 13-acetate; PRED, prednisone; RAPA, rapamycin; rev., reverse; sIL2R; soluble interleukin 2 receptor; TAC, tacrolimus; TDA, 2,2′:6′,2″-terpyridine-6,6″-dicarboxylic acid; TEC, renal tubular epithelial cells; TNF, tumor necrosis factor. Text in bold corresponds to the results of the current study*.

Our comparative *in vitro* study demonstrated that all ISD inhibited NK cell proliferation similar to T cell proliferation without affecting NK cell viability. The strongest inhibitor was MePRD. These results are in line with several other reports using IL2 or other cytokines, occasionally in combination, to induce NK proliferation (37, 38, 41–43, 48). Interestingly, IL15-induced NK cell proliferation was resistant to inhibition by MePRD (36); and inhibition by MePRD, RAPA, and MMF was more potent compared to calcineurin inhibitors (43). Accordingly, Ohata *et al* showed that inhibition by MPA was stronger compared to calcineurin inhibitors and methotrexate, without testing EVE/RAPA or corticosteroids (42); prednisolone inhibited renal tubular epithelial cell-induced NK cell proliferation more efficiently than TAC or EVE (37), and both MPA and RAPA inhibited NK cell proliferation more efficiently than CsA, especially proliferation of CD56^bright^ NK cells (38).

Nevertheless, Wang et al reported inhibition by CsA, in particular of the CD56^+^CD16^−^KIR^−^ NK subset, Kim et al showed strong dose-dependent inhibition by TAC (45), and Meehan et al that CsA was more potent than MPA, comparable to prednisolone (48). Thus, all ISD strongly inhibit NK cell proliferation with corticosteroids having the most potent effect, whereas conflicting results obtained with calcineurin inhibitors and MPA might not only stem from different stimulation protocols but also from a differential effect on CD56^bright^ compared to CD56^dim^ NK cells (see below). Most importantly, clinically applied ISD combinations completely abolished NK cell proliferation; exhibiting a cumulative effect. Finally, IVIg did not affect NK cell proliferation, in contrast to T cell proliferation which was significantly and dose-dependently inhibited.

The cytolytic and cytokine-producing activities of NK cells are highly regulated by a balance of activating and inhibitory receptors (63), therefore we examined the effect of ISD and IVIg on the phenotype of NK cells. In our hands, exposure to ISD for 24 h had only marginal effects on the expression of the majority of NK receptors tested. Expression of the activation markers CD25 and CD69; as well as of CD54, an adhesion molecule which is important for immune synapse formation during NK cytotoxicity, was reduced by several ISD. In addition, MePRD also slightly decreased the expression of the inhibitory receptor NKG2A and the activating receptor NKG2D. As to the molecular mechanism, caspases have been shown to be involved in the induction of CD69 and CD25 in human NK cells after activating receptor cross-linking (64). Therefore, we could speculate that some ISD interfere with these caspase pathways. No changes of expression were detected for NKp30 and NKp46, nor for DNAM-1 (CD226), the counter balance receptor TGIT (65) was not studied.

In contrast, the group of Mingari (36, 40) reported that MePRD significantly down-regulates the expression of NKp30 and NKp44, perforin and CD69 in a dose-dependent and reversible manner; NKG2D, and 2B4 were partially inhibited, whereas NKp46, NKp80, NTBA, KIR and NKG2A remained unchanged. Ohira et al. showed a reduction of NKp44, TRAIL, and CD69 by several ISD (MePRD>calcineurin inhibitors and RAPA>MPA) whereas NKp30, NKG2D, and CD132 expression was only inhibited by MePRD (43). A dose-dependent and selective inhibition of CD56^dim^ NK cells by CsA was observed in a IL2/IL15 7d proliferation assay while CD56^bright^ NK cells were relatively resistant, moreover, CsA reduced KIR, NKp44 and NKG2D expression, but increased NKp30 (41). In two other studies, CsA either had no major effect on the CD56^bright/dim^ balance (38) or led to a reduction of CD56^dim^ cells (42). In contrast, MPA and RAPA severely inhibited the outgrowth of CD56^bright^ NK cells, and the shift toward an activated NKG2A^+^KIR^−^NCR^+^ phenotype upon stimulation (38, 42). Finally, MPA reduced the expression of all activating NK cell receptors, including NKG2D, NKp30, NKp44, and NKp46 by more than 50% (42). The differences with our results are most likely explained by the short incubation time in our assays which was chosen to reflect the (twice) daily dosing of ISD in the clinics. IL2 was added to increase viability and functional “fitness” of NK cells; (however, controls without IL2 were also performed). Following this notion, long-term MPA incubation significantly compromised NK cell phenotype and function, whereas previously IL2-activated NK cells were rather resistant to short-term MPA treatment (52). Of note, IVIg had no effect on the phenotype of NK cells. In conclusion, surface receptor expression is not a likely explanation for the effect of the ISDs on NK cell function.

The effect of ISD on direct NK cytotoxicity using purified NK cells was the focus of several studies in the last decade with conflicting results (Table 2). Using the prototypic target cell line K562 we show here that direct NK cytotoxicity was impaired by MePRD and to a lesser extent by EVE, MPA, TAC and CsA (Table 1). Overnight exposure to a combination of TAC/CsA+MPA+MePRD suppressed NK cytotoxicity almost completely, in contrast to the combination of EVE/MPA causing only minimal inhibition. Our findings were complemented by assays measuring CD107a surface expression. Again MePRD and ISD combinations had a clear inhibitory effect whereas CsA, TAC, EVE ,and IVIg had no effect on degranulation, while MPA had only minor effects on CD56^bright^ NK cells. The selective inhibition of degranulation in the CD56^bright^ subpopulation may explain some of the contradictory reports in the literature regarding the effect of MPA on NK cells (38, 42, 43, 52). Nevertheless, the exact mechanisms of how different ISD as diverse as calcineurin inhibitors, antiproliferative agents and mTOR inhibitors interfere with NK cell degranulation remain elusive; potentially ISD interfere directly with signaling pathways involved in degranulation or with cross-communication pathways within the cell.

Corticosteroids have been previously shown to inhibit direct NK cytotoxicity (36, 37, 39, 43). In contrast, Demmers et al using renal tubular epithelial cells as targets (37), and Ohira et al using K562 target cells (43) observed that TAC, CsA, MPA and EVE/RAPA have no or little effect. Shin et al. reported a mild decrease of NK cell degranulation following treatment with CsA, TAC and MPA and a substantial reduction by MePRD (56). Furthermore, TAC was unable to prevent the lysis of mesenchymal stem cells by IL2/IL15-activated NK cells (53), but TAC significantly reduced NK killing of K562 and CEM target cells following culture in IL2 (45). Meehan et al showed a greater inhibition of NK degranulation and cytotoxicity by MPA than by MePRD and no effect of CsA (48). NK lysis of K562 and Daudi cells was also inhibited by 7d exposure to very high doses of MPA but not by CsA, TAC or MTX as reported by Ohata et al (42), whereas Brehm et al. demonstrated that K562 killing mediated by 24h IL2-stimulated NK cells was moderately susceptible to MPA at 30 times lower doses (52). In accordance, Eissens et al showed moderate inhibition of K562 killing by MPA and RAPA, but not by CsA (38). Intriguingly, Wang et al reported that cytotoxicity of CsA-cultured NK cells was even slightly increased (41).

Due to differences in the experimental settings, in particular the duration of *in vitro* NK cell culture, stimulation with different target cells, cytokines and concentrations, ISD concentrations and cytotoxicity assays (labeling, target cells, duration) it is hard to compare the results of these studies. Taking all data together we conclude that MePRD, when used alone or in combination, was the most potent inhibitor of degranulation and direct NK cytotoxicity, MPA and to a lesser degree EVE/RAPA also seem to be inhibitory, whereas calcineurin inhibitors (CsA/TAC) have only little or no effect.

The effect of ISD on ADCC was addressed using porcine endothelial cells coated with human anti-pig natural antibodies showing a major reduction by MePRD whereas TAC, MPA and EVE were less inhibitory. These results are in line with another report relevant to transplantation. Shin et al. showed a strong inhibition of degranulation and intracellular IFNγ using anti-HLA sera and allogeneic PBMC as targets, but cell lysis was not tested (56); furthermore, calcineurin inhibitors significantly decreased IFNγ production but had minor effects on degranulation, while RAPA and MPA had almost no effect. In addition, Rose et al. showed inhibition of ADCC by dexamethasone using a panel of CD20^+^ lymphoma cell lines and rituximab (55); dexamethasone also attenuated cetuximab-induced ADCC of carcinoma cell lines (57). In our study, inhibition of ADCC by MePRD was accompanied by a partial suppression of degranulation but did not correlate with CD16 expression. Therefore, the inhibitory mechanisms of TAC, EVE and MePRD might depend on different targets in the ADCC signaling pathway downstream of CD16.

Although IVIg did not reduce NK cell degranulation in our hands, treatment with IVIg led to a substantial dose-dependent decrease of direct NK cytotoxicity. In addition, IVIg was the strongest inhibitor of ADCC, even at doses used in replacement therapy to treat immunodeficiencies. These results are in line with previous reports showing that IVIg suppress NK cytotoxicity and ADCC (22, 66), almost equally effective as prednisolone (40). Finally, early reports by the groups of Good and Herberman had also shown inhibition of NK cytotoxicity by IVIg without providing a mechanism (44, 67); and more recently, Jacobi et al demonstrated suppression of K562 killing by NK cells, both *in vitro* and *ex vivo*, in neurological patients treated with high-dose intravenous IVIg, paralleled by the induction of degranulation and IFNγ release (54). Our data partially contradict these findings since we did not observe IVIg-induced degranulation, however, we confirmed IFNγ release at one chosen concentration of IVIg. One possible explanation for this discrepancy could be the use of an *ex vivo* whole blood assay by Jacobi et al as compared to our overnight treatment of purified NK cells. Cytokine induction by IVIg has been reported previously both *in vitro* and *in vivo* (54, 68, 69) and several technical aspects might influence IVIg-induced IFNγ production. First, the level of Ig aggregates in the IVIg preparations tend to increase during storage time; therefore our experiments were always conducted with fresh IVIg preparations. Second, the plastic materials used during the assays can also create artifacts in the stimulation levels of the different cells. Third, the anticoagulants used for *in-vitro* whole blood assays have been found to have an impact on the quantitative measurement of cytokines. The exact mechanisms have not yet been explored and are currently under investigation in our laboratory.

Intracellular IFNγ production by NK cells stimulated with IL2/IL12 and K562 cells was significantly decreased after overnight exposure to MePRD and calcineurin inhibitors, but not by MPA and EVE. Moreover, ISD combinations showed almost complete inhibition, with the exception of EVE/MPA. IFNγ secretion into culture supernatants was inhibited by all ISD, whereas IVIg slightly increased IFNγ. These data are in agreement with previous studies showing an inhibitory effect of ISD alone or in combination on cytokine production, especially IFNγ (38, 41–43, 45, 46, 48, 49, 52, 56, 58), and elevated IFNγ release by IVIg (54). In addition, we also observed a preferential impact of MPA on IFNγ production in CD56^bright^ NK cells (38, 52). In addition to our, several comparative studies point out that calcineurin inhibitors exert a particularly potent reduction of IFNγ production and release (58). Intriguingly, in one particular study CsA treatment induced even higher numbers of IFNγ-producing cells after IL12/IL18 stimulation, and a relative resistance of CD56^bright^ NK cells. However, this study missed to include target cell encounter (43). Nevertheless, an effect of calcineurin inhibitors on IFNγ production is expected, since they target the transcription factor NFAT, which drives cytokine gene expression.

The observation that some ISD affect cytotoxicity without affecting degranulation or affect cytotoxicity and not cytokine production warrants further studies of the molecular mechanisms. Although similar, the secretory pathways for cytokine and lytic granule content release by NK cells are regulated differently, in particular using different signaling cascades (70). It is of note that CD107a expression is a direct marker of degranulation and does not necessarily predict killing which depends on the effect of the granule content. Moreover, NK cytotoxicity includes pathways independent of lytic granules, involving death receptor ligands such as FasL and TRAIL (71). However, K562 cells are usually highly resistant to TNF-, FasL-, and TRAIL-induced apoptosis (72). Thus, CD107a and classical cytotoxicity assays measure different aspects of cellular cytotoxicity and they do not always show the same effect. Therefore, we speculate that ISD could have a different effect on CD107a expression and/or the content/polarization of the lytic granules thereby affecting their function differentially. The potential molecular mechanisms leading to the inhibition of NK cytotoxicity by IVIg are currently under investigation in our laboratory, in particular conjugate formation, granule polarization, Ca^2+^ mobilization, and signaling transduction pathways. As to the observed discrepancies with IFNγ depending on the assay performed, we also speculate that ISD affect different mechanistic pathways involved in the synthesis and/or release of this cytokine.

There are two further limitations of the current study to be considered. First, no analysis was performed in blood samples from patients under ISD or IVIg treatment. However, several such *ex vivo* studies from allogeneic bone marrow, lung, and mainly renal transplant recipients, essentially support the *in vitro* data discussed above. One early study showed that in contrast to CsA treatment alone, the combination of CsA or AZA with corticosteroids decreased both NK cytotoxicity and ADCC; monotherapy with corticosteroids led to a reduction of ADCC (73). NK cytotoxicity against different tumor cell lines was reduced in pediatric MePRD-treated bone marrow transplant recipients associated with a lower expression of NKp46 and NKp30 (40). One year after renal transplantation patients under TAC/MPA as compared to CsA/AZA had better preserved NK cell numbers and cytotoxicity (47). Moreover, the group of Falk analyzed the peripheral NK cell repertoire and cytokine production in renal transplant patients and found different results with calcineurin inhibitors and RAPA. Recently, increased numbers of NK cells were obtained from heart transplanted patients treated with EVE as compared to MMF, and these NK cells displayed dimished levels of inhibitory receptors (NKG2A, KIR3DL1) and increased degranulation in response to IL2/IL15 activation (51). Finally, NK cells in operationally tolerant renal transplant recipients off immunosuppression had decreased CD16, NKp46, perforin, granzyme A, and IFNγ expression associated with a strong impairment of cytotoxicity (50). This finding suggests the establishment of a pro-tolerogenic environment with impaired NK cell activity, and argue against an important role of NK cells in maintaining transplantation tolerance (11).

Second, several older and more recently introduced ISD were not examined. Azathioprine (74), methotrexate (42, 75, 76) and (hydroxy)chloroquine (77, 78) have been shown to impact NK cell phenotype and function. On the other hand, fingolimod (FTY720) increases NK cytotoxicity by up-regulation of NKp30, NKp44 and NKG2D expression (79). Furthermore, the new generation of anti-inflammatory biologicals such as tocilizumab (anti-IL6R) (80), anti-TNF drugs (81), B cell depleting monoclonal antibodies such as rituximab (82), or kinase inhibitors (83) can also influence NK cells.

**Figure 9:**
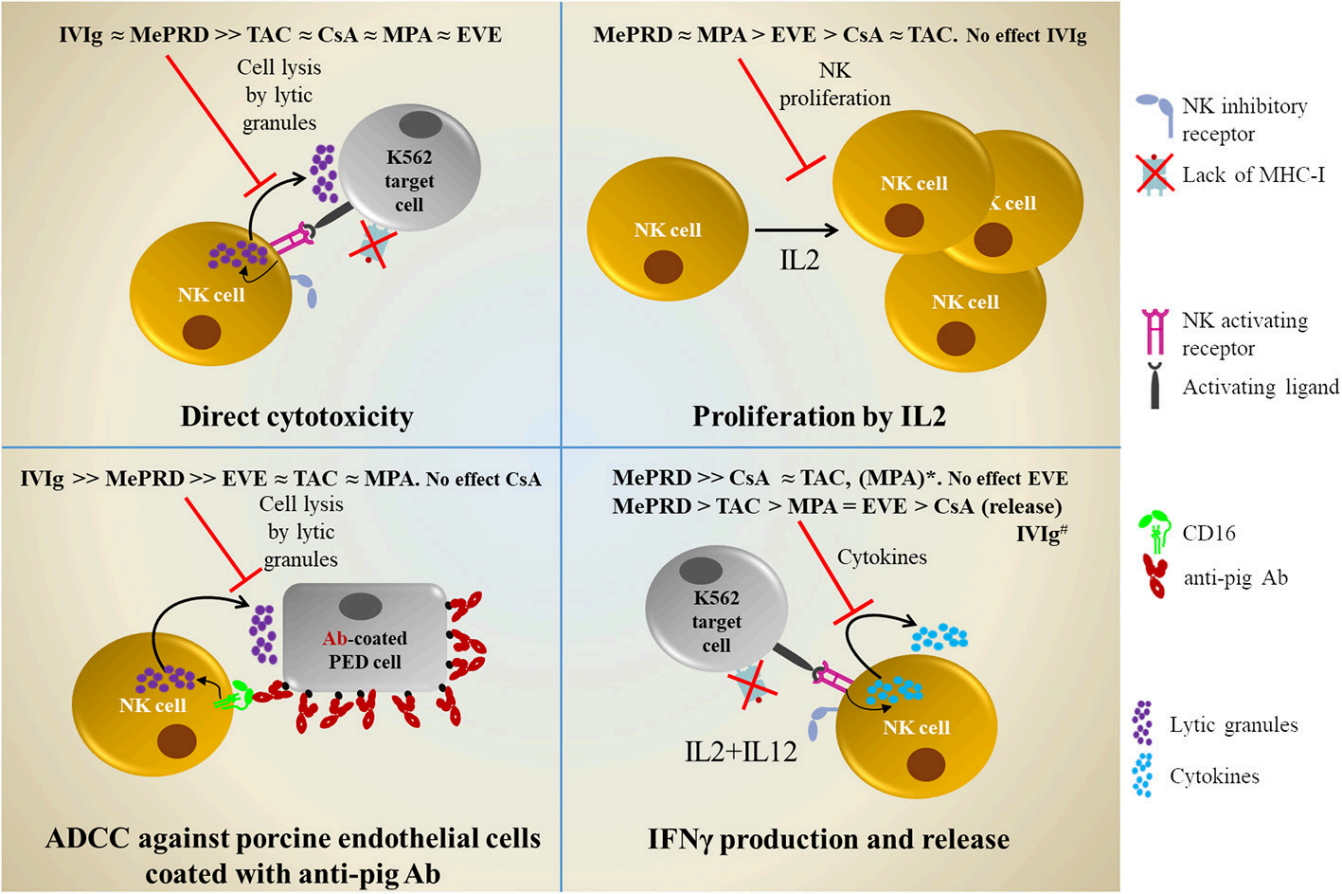
Comprehensive overview of the inhibitory effects of ISD and IVIg on NK cell functions. Direct cytotoxicity was tested following incubation overnight with IL2 (50 U/ml) and IL12 (0.5 ng/ml) in the presence or not of ISD and IVIg against K562 target cells using ^51^[Cr]-release and DELFIA cytotoxicity assays. Proliferation was tested following 5d incubation with IL2 (50 U/ml) by ^3^[H]-incorporation. ADCC was tested using porcine endothelial (PED) target cells coated with human anti-pig antibodies in ^51^[Cr]-release and DELFIA cytotoxicity assays. IFNγ production and release was tested following incubation overnight with IL2/IL12 followed by stimulation with K562 cells by intracellular flow cytometry and ELISA. *MPA only showed inhibitory effect on IFNγ production in the CD56^bright^ NK cell subpopulation. ^#^IVIg, enhances IFNγ production and release; opposite to ISD.

In summary, compared to CsA, TAC, MPA, and EVE, MePRD is the most potent inhibitor of NK cell effector functions, including proliferation, direct cytotoxicity, ADCC and IFNγ production. Combinations of CsA and TAC with MPA/and MePRD are also very potent inhibitors. Calcineurin inhibitors are potent inhibitors of IFNγ, but have only little effect on cytotoxicity, whereas EVE showed limited effects on NK cell functions. Furthermore, IVIg do not seem to affect NK cell functions responsible to control viral-infected cells, but significantly impair ADCC and direct NK cytotoxicity. A comprehensive overview is shown in Figure 9.

In conclusion, NK cell function should be preserved after transplantation in order to control viral infections and to provide tumor surveillance, *c.f*. Epstein Barr virus reactivation and posttransplant lymphoproliferative disease or graft vs. leukemia effects following hematopoietic stem cell transplantation; potentially also to promote transplantation tolerance. On the other hand, inhibition of NK cell function might be needed to prevent AbMR. Therefore, further clinical studies need to address the question which immunosuppressive and immunomodulatory drugs should be used, at what dose and when in order to achieve the best results. Indeed, based on the findings of the present study and the body of evidence currently available, RAPA/EVE ± MPA might represent a regimen without major negative impact on NK cell functions. The fact that MePRD and IVIg significantly block NK cytotoxicity, especially ADCC, has important implications for the treatment of AbMR, NK cell-based cancer immunotherapies, and monoclonal antibody therapies targeting cancer and immune cells.

## Data Availability

The datasets generated for this study are available on request to the corresponding author.

## Ethics Statement

Comite departemental d'ethique de medecine interne et medecine communautaire at University Hospitals Geneva. Protocols number 08-133 and 13-149.

## Author Contributions

AP participated in research design, performed the experiments and data analysis for ISD experiments, manuscript drafting. MP participated in research design, performed the experiments and data analysis in all experiments related to IVIg, finalizing the writing of the manuscript. NL performed the experiments and data analysis for ISD experiments. AR performed the experiments and data analysis of ADCC assays for ISD experiments. CK participated in research design, performed experiments and data analysis for ISD experiments. LG performed IFNγ detection assays by ELISA. RS discussion of results and critical revision of the manuscript. CV discussion of results and critical revision of the manuscript. GP participated in research design for IVIg experiments, discussion of the results, and critical revision of the manuscript. JS participated in research design, discussion of the results, writing, critical revision, and approval of the final submitted version of the manuscript.

### Conflict of Interest Statement

MP salary was partially paid by a unconditional research grant by CSL Behring AG; RS and CV are employees of CSL Behring AG. The remaining authors declare that the research was conducted in the absence of any commercial or financial relationships that could be construed as a potential conflict of interest.

## Supplementary Material

Figure S1 NK cell viability after culture with ISD. NK cells were incubated overnight in the presence of CsA (0.1 and 1.0 μg/ml), TAC (0.01 and 0.1 μg/ml), MPA (0.5 and 5 μg/ml), EVE (0.01 and 0.1 μg/ml), and MePRD (0.05, 0.5 and 5.0 μg/ml). 7AAD was added to the treated NK cells at a final concentration of 1 μg/ml, followed by flow cytometry. Data are presented as mean percentage of 7AAD- cells ± SD. Untreated NK cells kept overnight without stimulation were used as controls. CsA, cyclosporine A; EtOH, ethanol; EVE, everolimus; MePRD, methylprednisolone; MPA, mycophenolic acid; and TAC, tacrolimus.

Figure S2 Simultaneous determination of degranulation and intracellular IFNγ in NK cells treated with immunosuppressive drugs. Purified NK cells were analyzed for degranulation by staining for CD107a surface expression and intracellular IFNγ production by flow cytometry, following overnight culture with IL2 (50 U/ml) and IL12 (0.5 ng/ml) in the presence or absence of CsA (0.1–1 μg/ml), TAC (0.01–0.1 μg/ml), MPA (0.5–5 μg/ml), EVE (0.01–0.1 μg/ml), and MePRD (0.05–5 μg/ml) alone or in combination, and additional stimulation with K562 cells at a NK:K562 ratio of 1:1 for 3 h. Panels of flow cytometry dot-plots of a representative experiment are shown. Basal levels of CD107a and IFNγ are shown at the upper part of the figure. The percentage of NK cells expressing CD107a and/or IFNγ after stimulation in the presence or not of immunosuppressive drugs alone or in combination is indicated in the upper and right quadrants, respectively.

Table S1 Effect of immunosuppressive drugs on the expression of NK cell markers and receptors. PBMC were incubated with or without 50 U/ml IL2 (control); with 50 U/l IL2 plus CsA (0.1 μg/ml), TAC (0.01 μg/ml), MPA (5 μg/ml), EVE (0.0 1μg/ml), or MePRD (0.5 μg/ml) for 24 h. NK cell marker and receptor expression was analyzed by FACS. Data are shown as mean ± SD of 6 (for CD25, CD54, CD69, and CD16A) or 3 independent experiments using different donors. ANOVA with Dunnett's Multiple Comparison Test as post-test was used. *P*-values with statistical significance are indicated by ^*^ and † for % positive cells and MFIR, respectively; not statistically significant is indicated by (−).

## Abbreviations

AbMR: antibody-mediated rejection
ADCC: antibody-dependent cellular cytotoxicity
CsA: cyclosporine A
EVE: everolimus
HLA: human leukocyte antigen
IVIg: polyspecific soluble immunoglobulins
ISD: immunosuppressive drugs
MePRD: methylprednisolone
MFIR: geometric mean fluorescence intensity ratios
MPA: mycophenolic acid
NCR: natural cytotoxicity receptor
NK: natural killer
PBMC: Peripheral blood mononuclear cells
PED: porcine endothelial cell (D haplotype)
RAPA: rapamycin
SOT: solid organ transplantation
TAC: tacrolimus.
