# Supplementary figures and images for: Small-Molecule Immunosuppressive Drugs and Therapeutic Immunoglobulins Differentially Inhibit NK Cell Effector Functions in vitro
Source: Front Immunol. 2019 Mar 27;10:556. doi: 10.3389/fimmu.2019.00556 (PMC6445861; doi:10.3389/fimmu.2019.00556)

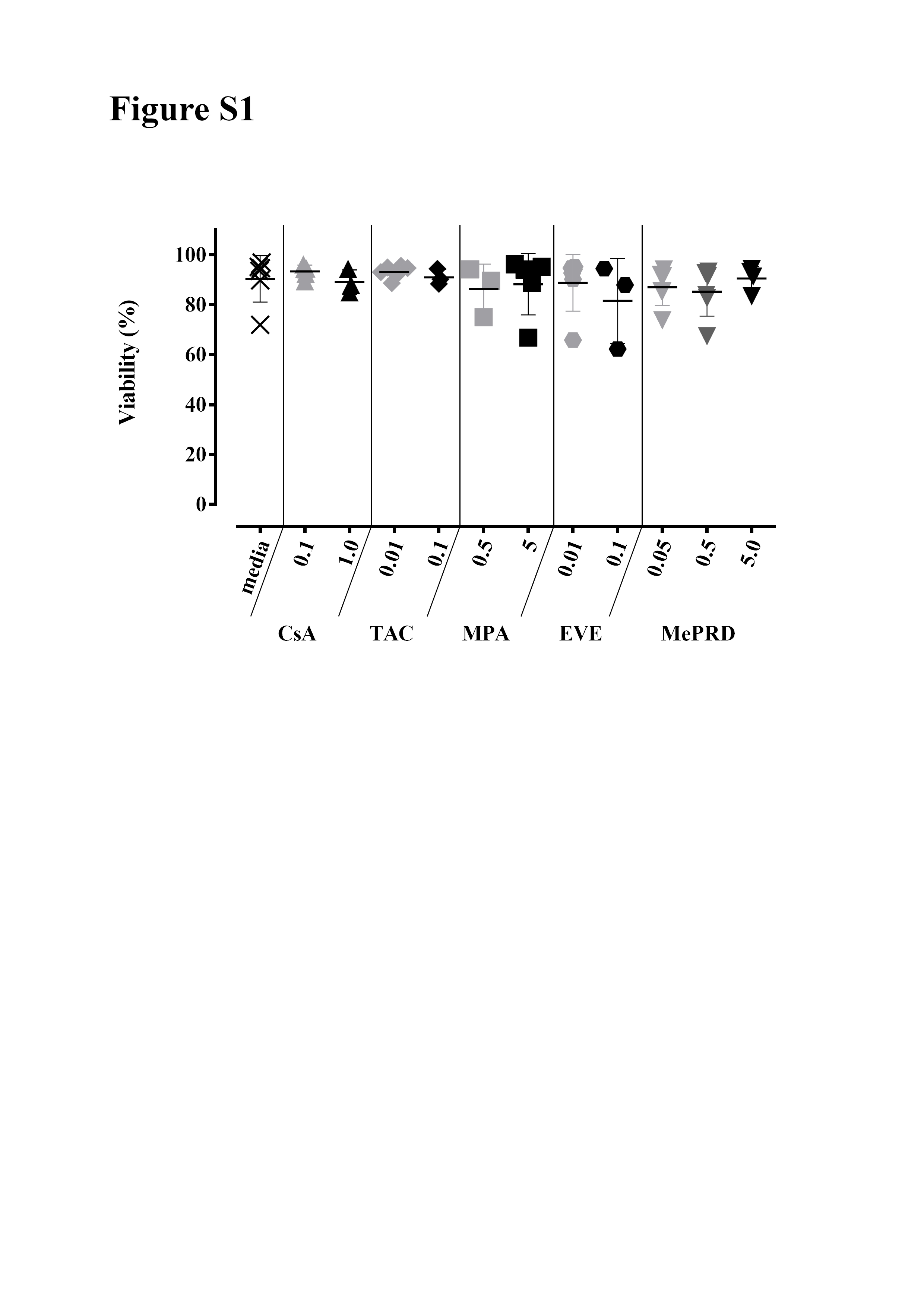

Supplement: Figure S1 — NK cell viability after culture with ISD. NK cells were incubated overnight in the presence of CsA (0.1 and 1.0 μg/ml), TAC (0.01 and 0.1 μg/ml), MPA (0.5 and 5 μg/ml), EVE (0.01 and 0.1 μg/ml), and MePRD (0.05, 0.5 and 5.0 μg/ml). 7AAD was added to the treated NK cells at a final concentration of 1 μg/ml, followed by flow cytometry. Data are presented as mean percentage of 7AAD- cells ± SD. Untreated NK cells kept overnight without stimulation were used as controls. CsA, cyclosporine A; EtOH, ethanol; EVE, everolimus; MePRD, methylprednisolone; MPA, mycophenolic acid; and TAC, tacrolimus. [file Image_1.JPEG]

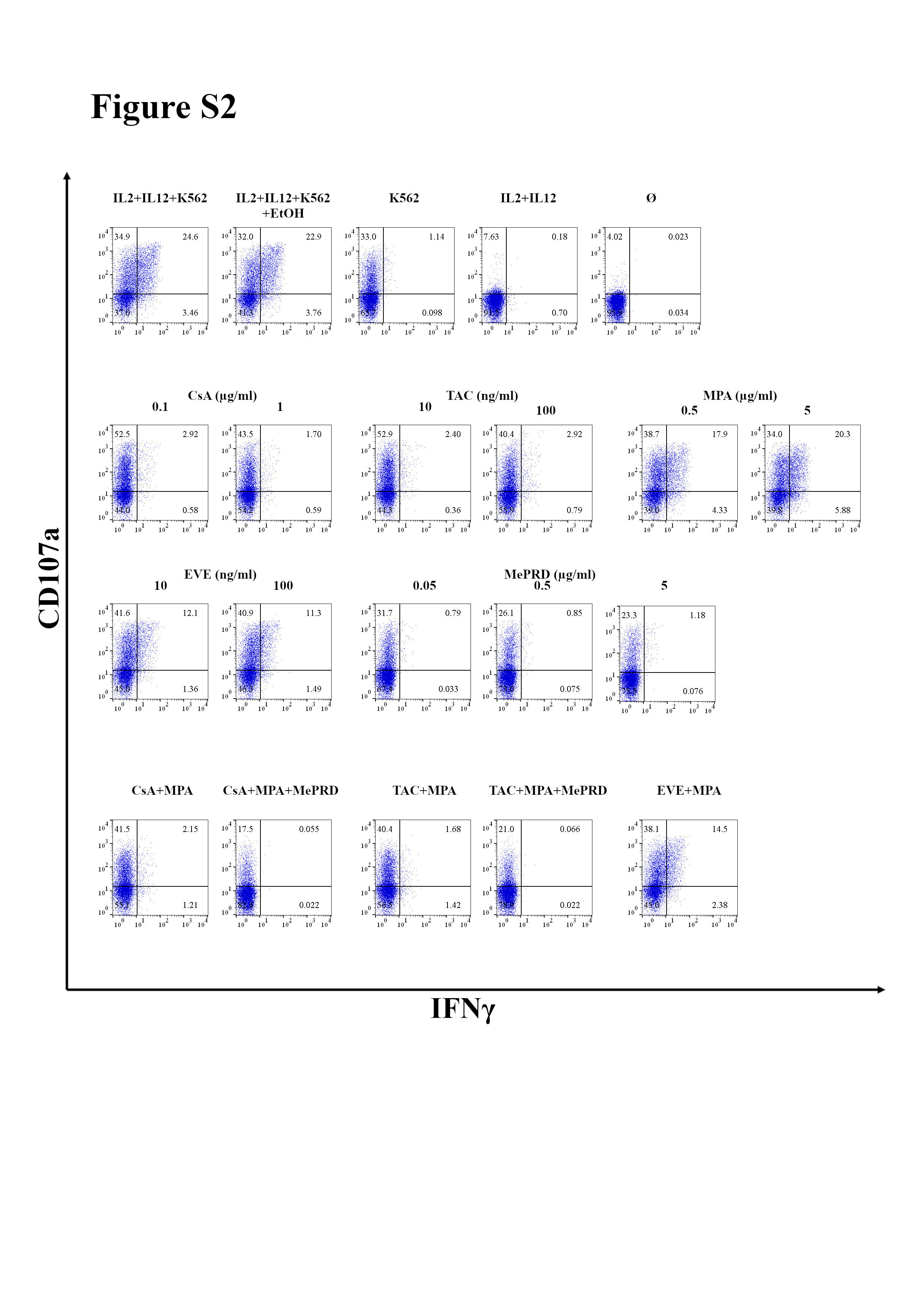

Supplement: Figure S2 — Simultaneous determination of degranulation and intracellular IFNγ in NK cells treated with immunosuppressive drugs. Purified NK cells were analyzed for degranulation by staining for CD107a surface expression and intracellular IFNγ production by flow cytometry, following overnight culture with IL2 (50 U/ml) and IL12 (0.5 ng/ml) in the presence or absence of CsA (0.1–1 μg/ml), TAC (0.01–0.1 μg/ml), MPA (0.5–5 μg/ml), EVE (0.01–0.1 μg/ml), and MePRD (0.05–5 μg/ml) alone or in combination, and additional stimulation with K562 cells at a NK:K562 ratio of 1:1 for 3 h. Panels of flow cytometry dot-plots of a representative experiment are shown. Basal levels of CD107a and IFNγ are shown at the upper part of the figure. The percentage of NK cells expressing CD107a and/or IFNγ after stimulation in the presence or not of immunosuppressive drugs alone or in combination is indicated in the upper and right quadrants, respectively. [file Image_2.JPEG]
