# Supplementary material for: Small-Molecule Immunosuppressive Drugs and Therapeutic Immunoglobulins Differentially Inhibit NK Cell Effector Functions in vitro
Source: Front Immunol. 2019 Mar 27;10:556. doi: 10.3389/fimmu.2019.00556 (PMC6445861; doi:10.3389/fimmu.2019.00556)
Supplement: Table S1 — Effect of immunosuppressive drugs on the expression of NK cell markers and receptors. PBMC were incubated with or without 50 U/ml IL2 (control); with 50 U/l IL2 plus CsA (0.1 μg/ml), TAC (0.01 μg/ml), MPA (5 μg/ml), EVE (0.0 1μg/ml), or MePRD (0.5 μg/ml) for 24 h. NK cell marker and receptor expression was analyzed by FACS. Data are shown as mean ± SD of 6 (for CD25, CD54, CD69, and CD16A) or 3 independent experiments using different donors. ANOVA with Dunnett's Multiple Comparison Test as post-test was used. P-values with statistical significance are indicated by * and † for % positive cells and MFIR, respectively; not statistically significant is indicated by (−). [file Table_1.DOCX]

**TABLES**

**Table Supplementary 1. Effect of immunosuppressive drugs on the expression of NK cell markers and receptors.** PBMC were incubated with or without 50U/ml IL2 (control); with 50U/l IL2 plus CsA (0.1μg/ml), TAC (0.01μg/ml), MPA (5μg/ml), EVE (0.01μg/ml), or MePRD (0.5μg/ml) for 24h. NK cell marker and receptor expression was analyzed by FACS. Data are shown as mean ± SD of 6 (for CD25, CD54, CD69 and CD16A) or 3 independent experiments using different donors. ANOVA with Dunnett's Multiple Comparison Test as post-test was used. *p* values with statistical significance are indicated by * and † symbols for % positive cells and MFIR, respectively; not statistically significant is indicated by (-).
